# Regulated phosphorylation of the K-Cl cotransporter KCC3 is a molecular switch of intracellular potassium content and cell volume homeostasis

**DOI:** 10.3389/fncel.2015.00255

**Published:** 2015-07-09

**Authors:** Norma C. Adragna, Nagendra B. Ravilla, Peter K. Lauf, Gulnaz Begum, Arjun R. Khanna, Dandan Sun, Kristopher T. Kahle

**Affiliations:** ^1^Department of Pharmacology and Toxicology, Boonshoft School of Medicine, Wright State UniversityDayton, OH, USA; ^2^Department of Pathology, Boonshoft School of Medicine, Wright State UniversityDayton, OH, USA; ^3^Department of Neurology, University of PittsburghPittsburgh, PA, USA; ^4^Department of Neurosurgery, Boston Children's Hospital and Harvard Medical School, Harvard UniversityBoston, MA, USA; ^5^Veterans Affairs Pittsburgh Health Care System, Geriatric Research, Educational and Clinical CenterPittsburgh, PA, USA; ^6^Manton Center for Orphan Disease Research, Children's Hospital Boston, Harvard UniversityBoston, MA, USA

**Keywords:** K-Cl cotransporters, KCC3, NKCC1, SPAK, cell volume homeostasis, regulatory volume decrease, cerebral edema, neurodegeneration

## Abstract

The defense of cell volume against excessive shrinkage or swelling is a requirement for cell function and organismal survival. Cell swelling triggers a coordinated homeostatic response termed regulatory volume decrease (RVD), resulting in K^+^ and Cl^−^ efflux via activation of K^+^ channels, volume-regulated anion channels (VRACs), and the K^+^-Cl^−^ cotransporters, including KCC3. Here, we show genetic alanine (Ala) substitution at threonines (Thr) 991 and 1048 in the KCC3a isoform carboxyl-terminus, preventing inhibitory phosphorylation at these sites, not only significantly up-regulates KCC3a activity up to 25-fold in normally inhibitory isotonic conditions, but is also accompanied by reversal of activity of the related bumetanide-sensitive Na^+^-K^+^-2Cl^−^ cotransporter isoform 1 (NKCC1). This results in a rapid (<10 min) and significant (>90%) reduction in intracellular K^+^ content (K_i_) via both Cl-dependent (KCC3a + NKCC1) and Cl-independent [DCPIB (VRAC inhibitor)-sensitive] pathways, which collectively renders cells less prone to acute swelling in hypotonic osmotic stress. Together, these data demonstrate the phosphorylation state of Thr991/Thr1048 in KCC3a encodes a potent switch of transporter activity, K_i_ homeostasis, and cell volume regulation, and reveal novel observations into the functional interaction among ion transport molecules involved in RVD.

## Introduction

Regulation of cell volume is critical for multiple essential cellular functions and organismal survival. Lacking a rigid cell wall, animal cells effectively combat excessive cell swelling or shrinkage, induced by perturbations in intracellular ion content or extracellular osmolality, by triggering concerted counter-responses termed regulatory volume decrease (RVD) or regulatory volume increase (RVI), respectively (Kregenow, 1971, 1981; Hoffmann and Dunham, 1995; Lauf and Adragna, 2000, 2012; Hoffmann et al., 2009). These mechanisms are highly regulated by signaling molecules that sense changes in intracellular ionic content and/or cell volume, and transduce these signals to the cell membrane to modulate the transport of ions and/or organic osmolytes via the stimulation or inhibition of ion transporters, pumps, and channels (Hoffmann and Dunham, 1995). The *SLC12A* family of cation-Cl^−^ cotransporters [including the Na^+^-K^+^-2Cl^−^ cotransporter isoform 1 NKCC1 and the K^+^-Cl^−^ cotransporters (KCCs), such as KCC3], the Na^+^/H^+^ exchangers (e.g., NHE1), the Na^+^/K^+^ pump, and volume-regulated anion channels (VRACs), are important plasmalemmal mediators of ion transport in RVI and RVD (Hoffmann and Dunham, 1995; Lauf and Adragna, 2000, 2012; Hoffmann et al., 2009).

K^+^-Cl^−^ cotransport was first identified as a swelling- and thiol-activated K^+^ efflux pathway in low-K^+^ sheep red blood cells (Dunham et al., 1980; Lauf and Theg, 1980). The four KCC isoforms (KCC1-4) utilize energetically favorable, outwardly-directed K^+^ gradients to drive the extrusion of Cl^−^ across the plasma membrane. As such, they serve as important determinants of both intracellular K^+^ and Cl^−^ content, which are important for cell volume regulation and other essential functions depending on cell type (e.g., epithelial transport and neuronal excitability) and KCC isoform (Lauf and Adragna, 2012). The physiological importance of the swelling-activated KCCs, and in particular KCC3 (*SLC12A6*), is clearly illustrated by the phenotypes that result from the knockout of the genes encoding these molecules in mouse (Boettger et al., 2002; Delpire and Mount, 2002), and their mutations in humans (Howard et al., 2002).

Loss-of-function mutations in human KCC3 cause the autosomal recessive disease Andermann syndrome (OMIM # 218000), characterized by agenesis of the corpus callosum, peripheral neuropathy (ACCPN), and seizures (Delpire and Mount, 2002; Howard et al., 2002; Uyanik et al., 2006). KCC3 KO mice recapitulate the human ACCPN phenotype (Howard et al., 2002; Boettger et al., 2003) and studies in these mice have shown that disease pathogenesis is associated with neuroglial swelling and subsequent neurodegeneration resulting from impaired RVD (Byun and Delpire, 2007). Dysfunction of the volume-regulated KCCs has been implicated in other cell volume-related pathologies (Adragna et al., 2004, 2006; Adragna and Lauf, 2007), such as RBC dehydration in sickle cell disease (Brugnara et al., 1986; Brugnara, 1993, 1995, 2001; Joiner, 1993; Lew and Bookchin, 2005) and inner ear cell degeneration in deafness (Boettger et al., 2003).

Despite their physiological importance, the regulation of the KCCs is not fully understood, though serine-threonine kinases/phosphatases have long been known to play an essential role (Jennings and Al-Rohil, 1990). Phosphorylation inhibits the KCCs, while dephosphorylation has the opposite effect (Dunham et al., 1980; Altamirano et al., 1988; Jennings and Schulz, 1991; Lytle and Forbush, 1992; Haas and Forbush, 2000; Adragna et al., 2004). KCC1, KCC3, and KCC4 are inactive in isotonic conditions but briskly activated in response to hypotonic, low Cl^−^ cell-swelling conditions by a regulatory mechanism that is evolutionarily-conserved (Haas and Forbush, 2000; Adragna et al., 2004; Strange et al., 2006). KCC activation by hypotonic cell swelling is prevented by calyculin A, an inhibitor of protein phosphatase 1A (PP1) and PP2, further demonstrating the importance of phosphorylation for KCC regulation (Lauf and Adragna, 2000).

Two threonine (Thr) residues in human KCC3a, Thr991, and Thr1048, were recently shown to undergo rapid dephosphorylation in response to hypotonic low Cl^−^ (swelling/activating) conditions in HEK293 cells and human RBCs (Rinehart et al., 2009; De Los Heros et al., 2014); physiologically, these sites are phosphorylated in isotonic (inhibitory) conditions. Homologous sites (i.e., “site 1” and “site 2”) are phosphorylated in all human KCCs, including KCC2, and double alanine substitution at these residues, preventing phosphorylation, result in constitutively-active KCC2 and KCC3 (Rinehart et al., 2009; De Los Heros et al., 2014). The with no lysine [K] (WNK)-regulated STE20/SPS1-related proline/alanine-rich kinase (SPAK) and oxidative stress responsive kinase-1 (OSR1) kinases, known to regulate the activity of the KCCs in oocytes (e.g., Kahle et al., 2005; Gagnon et al., 2006), were recently shown to directly phosphorylate site 2 in the KCCs, but not site 1 (De Los Heros et al., 2014). The kinase that directly phosphorylates site 1 in the KCCs has not yet been identified (Rinehart et al., 2009).

Despite the detailed *biochemical* characterization of the swelling-induced KCC3 Thr991/Thr1048 dephosphorylation mechanism, the *cellular physiology* of this event has not been systematically explored. Here, we utilized unidirectional net ion flux uptake/loss assays under zero-trans conditions, to measure intracellular K^+^ (K_i_) content and uptake of ^85^Rb, and cell volume analysis in two isogenic pairs of human epithelial cell lines (HEK-293) engineered with doxycycline-inducible expression of wild type KCC3 (KCC3 WT) or KCC3 Thr991Ala/Thr1048Ala (i.e., “KCC3 AA,” preventing inhibitory phosphorylation), on (1) KCC3 transport activity; (2) the activity of other key molecules involved in cell volume homeostasis [e.g., NKCC1 and the Na^+^/K^+^ pump (herein termed “NKP”)]; (3) K_i_; and (4) cell volume and RVD in conditions of hypotonic stress.

## Materials and methods

### Chemicals

Chemicals from Thermo Fisher Scientific (Fair Lawn, NJ) were: Tris (hydroxymethyl) aminomethane (Tris) free base, 3-morpholin-4-ylpropane-1-sulfonic acid (MOPS), sodium chloride (NaCl), potassium chloride (KCl), magnesium chloride (MgCl_2_), sodium hydroxide (NaOH), sucrose, D-glucose, perchloric acid, 70% (PCA), and bicinchoninic acid (BCA) protein assay reagents. Magnesium gluconate was from Sigma-Aldrich (St. Louis, MO). 4-(2-hydroxyethyl)-1-piperazine ethane sulfonic acid (HEPES) free acid, and anhydrous calcium chloride (CaCl_2_), were from J.T.Baker Chemical Co (Center Valley, PA). Rubidium chloride (RbCl), 99.8% (metals basis), and amidosulfonic acid (sulfamic acid, S), 99.99% (metals basis) were purchased from Alfa Aesar (Ward Hill, MA); N-methyl D-glucamine (NMDG) from Fluka Biochemika (St. Louis, MO); cesium chloride (CsCl) and calcein-AM from Life technologies (Carlsbad, CA) and calcium gluconate from Acros Organics (NJ). Ouabain octahydrate was purchased from Calbiochem (San Diego, CA), furosemide and bumetanide from Sigma-Aldrich (St. Louis, MO), 4-[(2-Butyl-6,7-dichloro-2-cyclopentyl-2,3-dihydro-1-oxo-1H-inden-5-yl)oxy]butanoic acid (DCPIB), 1,2-Bis(2-aminophenoxy) ethane-N,N,N′,N′-tetra acetic acid (BAPTA) from Tocris Bioscience (Bristol, UK), tetra ethyl ammonium (TEA) from Abcam (Cambridge, MA), clofilium tosylate from Enzo life sciences (Farmingdale, NY), and 2,4-dichloro-N-isopropyl-N-(2-isopropylaminoethyl)benzenesulfonamide (RN-1734) and Ruthenium Red (RR) from Santa Cruz Biotechnology (Santa Cruz, CA).

### Solutions

The solution compositions for the different steps in the flux protocol are as follows (with all salt concentrations in mM). Initial wash: 300 mOsM balanced salt solution (BSS-NaCl) (20 HEPES-Tris, 5 KCl, 2 CaCl_2_, 1 MgCl_2_, 10 glucose, 135 NaCl, pH 7.4, 37 °C) or BSS-NaS (20 HEPES-Tris, 5 K^+^ sulfamate, 2 Ca gluconate, 1 Mg gluconate, 10 glucose, 135 NaS, pH 7.4, 37 °C). Pre-incubation/equilibration: BSS-NaCl-BSA (bovine serum albumin) (300 mOsM BSS-NaCl + 0.1 % BSA, pH 7.4, 37 °C) or BSS-NaS-BSA (300 mOsM BSS-NaS + 0.1 % BSA, pH 7.4, 37 °C). Flux (in mM): 300 mOsM BSS-RbCl-BSA (20 HEPES-Tris, 10 RbCl, 2 CaCl_2_, 1 MgCl_2_, 10 glucose, 0.1 % BSA, 135 NaCl, pH 7.4, 37 °C) or BSS-RbS-BSA (20 HEPES-Tris, 10 Rb^+^ sulfamate, 2 Ca gluconate, 1 Mg gluconate, 10 glucose, 0.1 % BSA, 135 NaS, pH 7.4, 37 °C). Final wash: 300 mOsM containing 10 MOPS-TrisMgCl_2_, pH 7.4, 37 °C (Supplementary Table 2).

Ions were extracted for 15 min at 4 °C with 5 % perchloric acid (PCA) and measured by atomic absorption spectrophotometry in a Perkin Elmer 5000, as described elsewhere (Adragna et al., 2002; Zhang et al., 2003). Total protein was determined by protein extraction with 1M NaOH and measured with the BCA protein assay as previously described (Di Fulvio et al., 2001).

### Cell line construction and culture

WT or AA (Thr991Ala/Thr1048Ala) human KCC3a cDNAs harboring an N-terminal *myc* epitope (gifts of P. Gallagher, Yale University), under the expression control of tetracycline, were introduced as single copies into HEK-293 (Tet-On) cells using the Flp-In™ T-REx™ System as described elsewhere (Rinehart et al., 2009) (Supplementary Figure 2). Doxycycline, a tetracycline derivative, was used to induce gene expression per manufacturer's directions (Life Technologies-Invitrogen, Grand Island, NY 14072). Cells were cultured in Dulbecco's Modified Eagle Medium (DMEM) with high glucose (Life Technologies, Catalog No. 11965-092), supplemented with 10 % tetracycline-free fetal bovine serum (FBS) (Clontech, Catalog No. 631106), 100 units/mL penicillin, 100 μg/mL streptomycin (Hyclone, Catalog No. SV30010) and 10 μg/mL blasticidin S hydrochloride (Santa Cruz, Catalog No. sc-204655), and maintained in a humidified incubator with 5 % CO_2_ at 37 °C. For live cell imaging experiments, viable cells (0.2 × 10^6^ cells/well) were plated on poly-D-Lysine coated glass coverslips (22 × 22 mm) in 6-well plates. To induce KCC3 (WT or AA) expression, cultures were treated with 1 μg/mL doxycycline for up to 16 h.

### Ion flux studies

Ion fluxes were measured according to previously published protocols (Adragna et al., 2002; Zhang et al., 2003) with some modifications. Cells were grown to 60 % confluence in 12-well plates; KCC3 was expressed by overnight doxycycline (0–1 μg/ml) induction. ^85^Rb^+^ uptake or influx and intracellular ^39^K^+^ (K_i_) content were determined under zero-trans conditions as follows. WT- or AA-transfected cells were removed from the incubator; culture medium was aspirated, washed three times with 1 ml 300 mOsM BSS-NaCl or BSS-NaS and then equilibrated in BSS-NaCl-BSA or BSS-NaS-BSA for 10 min. Thereafter, supernatants were removed and then exposed to pre-warmed flux media 300 mOsM BSS-RbCl-BSA or BSS-RbS-BSA during pre-determined time points (0–15 min). ^85^Rb^+^ was used as a ^39^K^+^ congener (Lauf, 1983; Delpire and Lauf, 1991). Thereafter, cells were washed with washing solution to block Rb^+^ influx and K^+^ loss. Cellular Rb^+^ and K^+^ were extracted as described above. Intracellular K^+^ was simultaneously measured with a Na^+^-K^+^ lamp by absorption and Rb^+^ uptake with ^85^Rb^+^ by flame emission spectrophotometry in a Perkin-Elmer 5000 Atomic Absorption spectrophotometer (Perkin-Elmer, Boston MA). Total protein was determined by the BCA assay as described above. Rb^+^ uptake as a function of time and K_i_, (nanomoles per milligram protein) as a function of time were calculated for each well as described elsewhere (Adragna, 1991). Ouabain and bumetanide at final concentrations of 0.1 mM and 10 μM, respectively, were added to the flux media to block the Na^+^/K^+^ pump (NKP) and Na^+^-K^+^-2Cl^−^ cotransporter (NKCC), respectively. NKP-mediated Rb^+^ influx was calculated as the ouabain-sensitive flux (Cl - ClO) and NKCC as the ouabain-insensitive but bumetanide-sensitive (ClO - ClOB) Rb^+^ influx in Cl^−^ media, The Cl-dependent Rb^+^ influx (KCC) is the calculated difference between the Rb^+^ influx in Cl^−^ and S media, both containing ouabain and bumetanide (ClOB - SOB) (Supplementary Tables 1, 2).

### Cell volume measurements

Cell volume change was determined using calcein as a marker of intracellular water volume, as established previously (Lenart et al., 2004). Briefly, cells on coverslips were incubated with 0.5 μM calcein-AM for 30 min at 37 °C. The cells were placed in a heated (37 °C) imaging chamber (Warner Instruments, Hamden, CT) on a Nikon Ti Eclipse inverted epifluorescence microscope equipped with perfect focus, a 40X Super Fluor oil immersion objective lens, and a Princeton Instruments MicroMax CCD camera. Calcein fluorescence was monitored using a FITC filter set (excitation 480 nm, emission 535 nm, Chroma Technology, Rockingham, VT). Images were collected every 60 s with MetaFluor image-acquisition software (Molecular Devices, Sunnyvale, CA) and regions of interest (~20–30 cells) were selected. Baseline drift resulting from photo bleaching and dye leakage was corrected as described previously (Lenart et al., 2004). The fluorescence change was plotted as a function of the reciprocal of the relative osmotic pressure and the resulting calibration curve applied to all subsequent experiments as described previously (Lenart et al., 2004). The HEPES-buffered isotonic solution contained (in mM) 100 NaCl, 5.4 KCl, 1.3 CaCl_2_, 0.8 MgSO_4_, 20 HEPES, 5.5 glucose, 0.4 NaHCO_3_, and 70 sucrose (pH 7.4) with 310 mOsM determined using an osmometer (Advanced Instruments, Norwood, MA). Anisosmotic solutions (150, 280, and 312 mOsM) were prepared by removal or addition of sucrose to the above solution.

### Statistical analysis

The analysis of two samples in each or for independent experiments was done by paired or unpaired *t*-test. Multiple intergroup differences in each or for independent experiments was conducted by One-Way analysis of variance (ANOVA) or Kruskal–Wallis One-Way-ANOVA test followed by either, paired or unpaired *t*-test, Wilcoxon signed-rank test, two-sample *t*-test or Wilcoxon rank-sum test. A *p* < 0.05 was used as the criterion of statistical significance. Except where indicated, all values were obtained from three independent experiments in which at least triplicate samples were assayed.

## Results

### An isogenic cell line system with inducible expression of wild type or constitutively-dephosphorylated KCC3 (Thr991Ala/Thr1048Ala)

We constructed HEK-293 epithelial cell lines with doxycycline-regulated expression of either N-terminal myc-tagged wild type human KCC3a (“KCC3 WT”) or KCC3a Thr991A/Thr1048A (“KCC3 AA”) inserted as a single copy, as described in Materials and Methods. In the absence of doxycyline, KCC3 WT or AA is not expressed (Supplementary Figure 1). However, in the presence of doxycycline (0.1 μM), anti-myc antibody revealed robust expression of myc-tagged KCC3 WT or KCC3 AA in 1.5 h (Supplementary Figure 1). KCC3 expression and localization was similar across multiple clonal cell lines of both KCC3 WT and KCC3 AA (data not shown). Consistent with previous reports (Rinehart et al., 2009; De Los Heros et al., 2014), KCC3 in isotonic conditions was phosphorylated at both Thr991 and Thr1048 (Supplementary Figure 1), as revealed using phospho-specific antibodies raised against these residues (De Los Heros et al., 2014). Hypotonic low Cl^−^ conditions that stimulate KCC3 activity elicited dephosphorylation at Thr991 and Thr1048 in KCC3 WT (data not shown), and mutation of these residues to alanine (A) in KCC3 AA prevented phosphorylation at each site (Supplementary Figure 1).

### Constitutive KCC3 Thr991/Thr1048 dephosphorylation potently stimulates KCC3 and is associated with a reversal of NKCC1 activity in flux conditions

As a reminder ^85^Rb^+^ (a K^+^ congener) was used in uptake assays and ^39^K^+^ under zero-trans conditions for K_i_ content determinations to measure the activities of KCC, NKCC, and NKP (see Materials and Methods for details).

It is also important to stress that only one technique is used to determine simultaneously (in the same cells and at the same time) both Rb^+^ influx and K_i_ content, which are measured by atomic absorption (K^+^) and emission (Rb^+^) spectrophotometry. Briefly, the technique consists of the following main steps: (1) Cells plated on 12-well plates; (2) Culture medium removal from wells at confluence; (3) Overnight incubation with doxycycline (Induction); (4) Washing of cells 3×; (5) Equilibration with solutions of similar composition as the flux solutions containing K^+^ but not Rb^+^ (Preincubation); (6) Flux with solutions containing Rb^+^ but not K^+^ (Flux); (7) Washing of cells 3 times; (8) Further processing for cations and protein determinations (See Materials and Methods for more details and Supplementary Tables 1, 2).

This technique, which has been developed several decades ago, allows to measure simultaneously unidirectional fluxes under 0-trans conditions (Rb^+^ influx and K^+^ efflux) without the use of radioisotopes (Lauf, 1983; Delpire and Lauf, 1991), and was applied when measuring simultaneously K^+^ loss and Rb^+^ influx through the IK channel of lens epithelial cells (Lauf et al., 2008). This technique also allows measuring K_i_ in a simplified manner, especially in KCC3 AA cells, because K_i_ loss is of such magnitude that the signal to noise ratio is large enough to produce accurate data.

Rb^+^ influx through KCC is defined as the difference in Rb^+^ influx between Cl-containing and Cl-free medium with sulfamate (S) as Cl^−^ replacement, in the presence of both ouabain (a NKP inhibitor) and bumetanide (a NKCC inhibitor); Rb^+^ influx through NKCC is defined as the ouabain-insensitive and bumetanide-sensitive Rb^+^ influx in Cl-containing media; and Rb^+^ influx through NKP is defined as the ouabain-sensitive Rb^+^ influx in Cl-containing medium (Supplementary Tables 1, 2).

**Figure 1:**
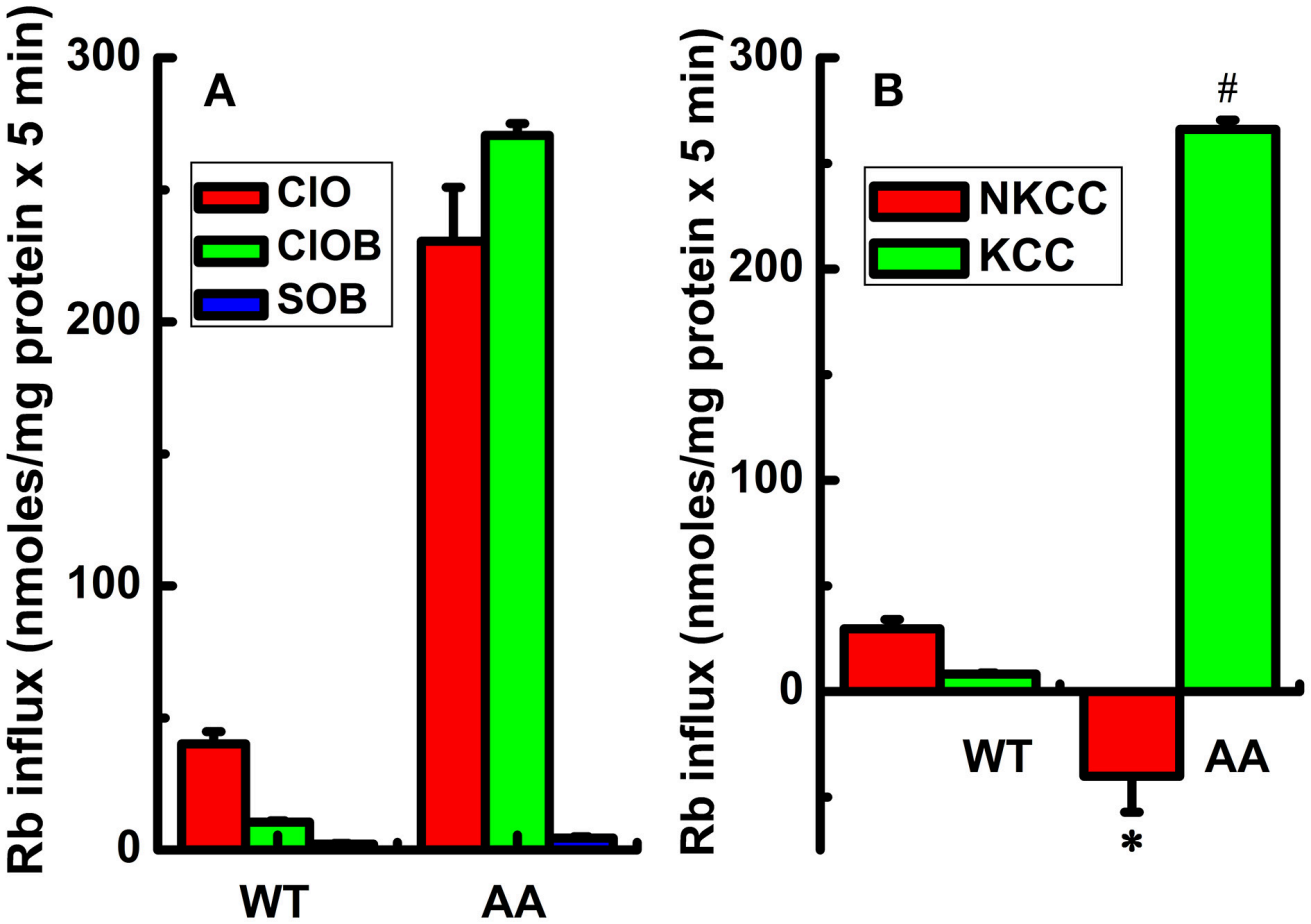
**Constitutive KCC3 Thr991/Thr1048 dephosphorylation stimulates KCC3 activity and is accompanied by a reversal of NKCC1 activity**. Rb^+^ influx assays in isogenic cell lines harboring doxycycline-inducible expression of either KCC3 wild type (WT) or non-phosphorylatable KCC3 Thr991A/Thr1048A (AA) were performed as described in Methods. **(A)** Rb^+^ influx was assessed in the following media: Cl^−^ + 0.1 mM ouabain (ClO, red), Cl^−^ + (ouabain + 10 μM bumetanide) (ClOB, green), S + (ouabain + bumetanide) (SOB, blue) after induction of the indicated proteins (see Methods for details). **(B)** Calculated Rb^+^ flux through NKCC and KCC as the Cl-dependent, ouabain-insensitive, bumetanide-sensitive Rb^+^ influx (ClO - ClOB), and Cl-dependent, ouabain and bumetanide-insensitive Rb^+^ influx (ClOB - SOB), respectively. Calculation of NKCC in AA cells after induction resulted in a negative value due to a larger Rb^+^ influx in ClOB than in ClO, i.e., CLO – CLOB = -NKCC (see Results and Discussion for further details). Flux time, 5 min; *n* = 9 individual determinations for WT cells and *n* = 4 individual determinations for AA cells. Total number of independent experiments *N* = 4 for WT and *N* = 5 for AA cells. Results were similar among different cell clones for both WT and AA. ^*^p < 0.005; ^#^*p* < 0.0005; data represent the mean ± SEM values. Two-sample *t*-test was employed to determine the statistical significance of the differences between WT and AA, as indicated.

Induction of KCC3 WT in isotonic conditions did not elicit a significant KCC-mediated Rb^+^ influx. For instance, in control cells (i.e., cells not induced with doxycycline) the Rb^+^ influx was 10.6 ± 1.8 nmol/mg protein × 5 min, whereas in cells induced with doxycycline (shown in Figure 1B), it was 12.4 ± 0.9 nmol/mg protein × 5 min, *n* = 12 individual determinations from 4 independent experiments, consistent with the presence of inhibitory phosphorylation of KCC3 at Thr991 and Thr1048 (Supplementary Figure 1). Likewise, in KCC3 WT cells, NKCC was 30.2 ± 7.4 in non-induced cells and 29.7 ± 6.0 nmol/mg protein × 5 min in induced cells (shown in Figure 1B), *n* = 12 individual determinations from 4 independent experiments.

In contrast, induction of KCC3 AA caused a significant increase in Rb^+^ influx in Cl^−^ and ouabain +/− bumetanide media (ClO and ClOB), which was almost completely inhibited in Cl-free medium (SOB) (Figure 1A), resulting in a greater than ~10-fold elevation of KCC activity [non-induced cells, 19.1 ± 0.42 nmol/mg protein × 5 min and in induced cells (shown in Figure 1B), 266.1 ± 2.2 nmol/mg protein × 5 min, *n* = 12 individual determinations from 4 independent experiments, *p* < 0.00001]. This effect was associated with a ~5-fold *decrease* in NKCC activity; note the *negative* NKCC value in Figure 1B that was calculated as the difference between ClO - ClOB Rb^+^ influx (plotted in Figure 1A) [non-induced, 108.5 ± 3.2 and in induced (shown in Figure 1B), −40.1 ± 8.5 nmoles/mg protein × 5 min, *n* = 12 individual determinations from 4 independent experiments, *p* < 0.0005].

**Figure 2:**
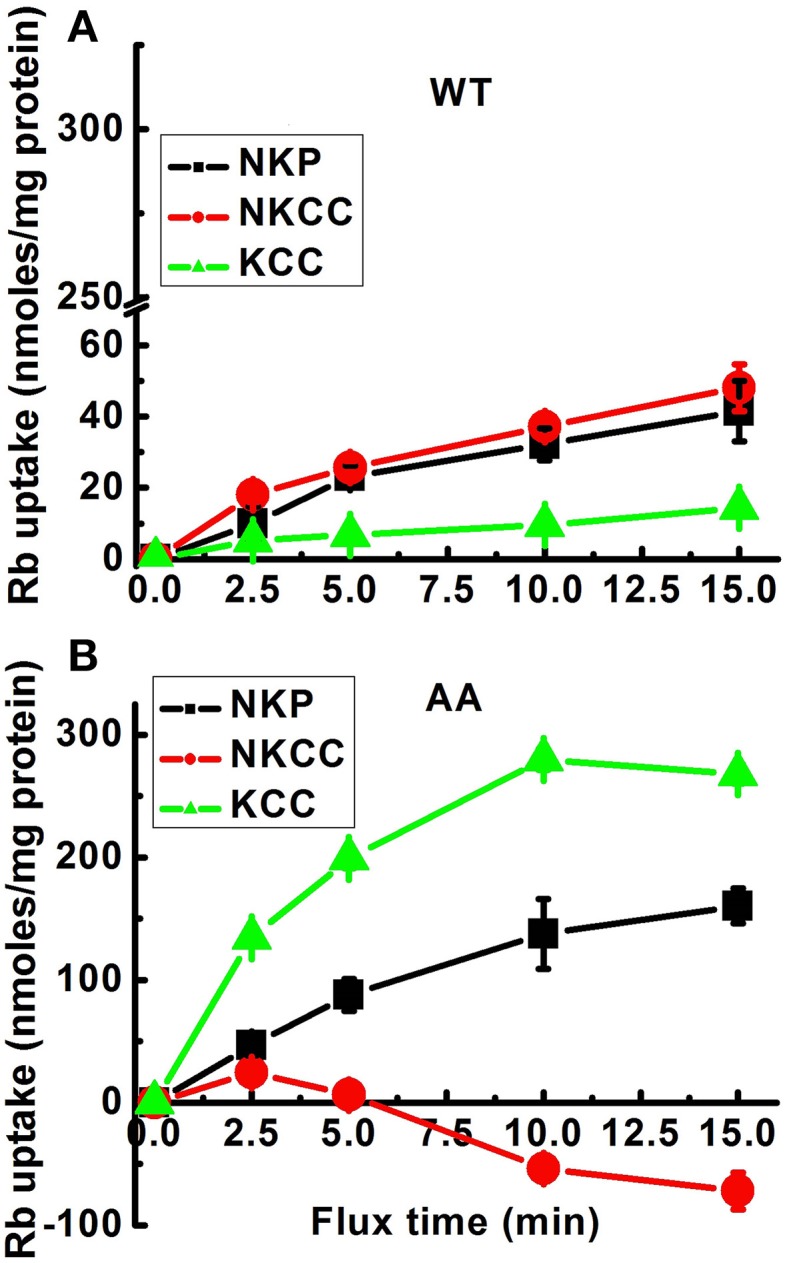
**Effect of constitutive KCC3 Thr991/Thr1048 dephosphorylation on KCC3, NKCC1, and NKP activity as a function of flux time**. Rb^+^ influx assays in isogenic cell lines harboring inducible expression of either KCC3 wild type (WT) **(A)** or KCC3 Thr991A/Thr1048A (AA) **(B)** were performed as described in Methods. Rb^+^ uptake was determined in the following media: total uptake, Cl^−^ alone (Cl), Cl^−^ + ouabain (ClO), Cl^−^ + ouabain + bumetanide (ClOB), S + ouabain + bumetanide (SOB). NKP, NKCC, and KCC represent the Rb^+^ influx through the corresponding transporters. Data for WT **(A)** were represented in the same scale as for AA **(B)** cells to show the quantitative difference in Rb^+^ uptake between these two types of cells. Shown are 2 independent experiments, *n* = 9 individual determinations for WT cells and *n* = 4 individual determinations from a single experiment for AA cells. Total number of independent experiments, *N* = 3 for both WT and AA cells. Data represent the mean ± SEM values. See legend to Figure 1 for further details.

**Figure 3:**
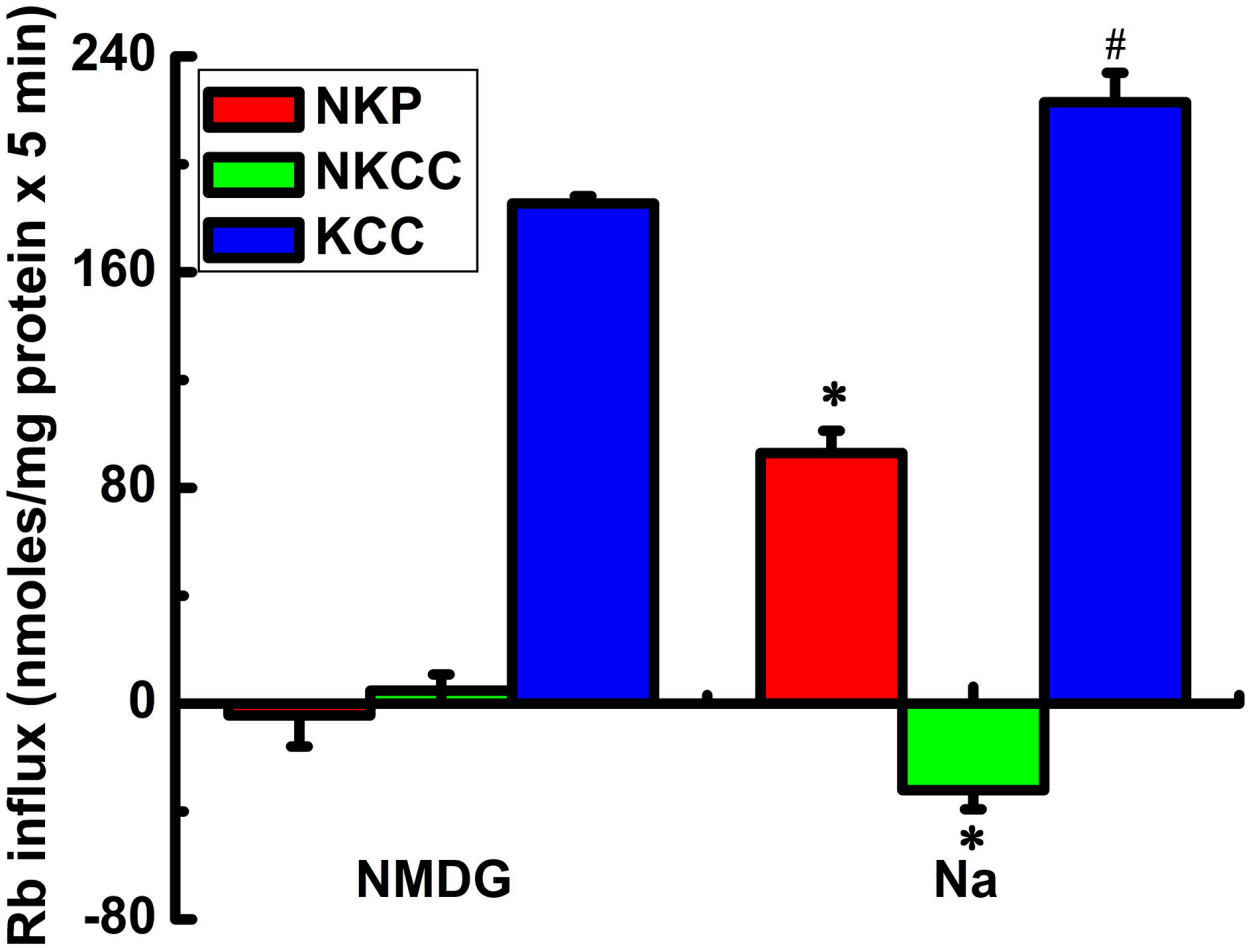
**Rb^+^ influx via KCC3, NKCC1, and NKP in Na^+^ and N-methyl-D-glucamine (MDG) medium**. Rb^+^ influx through the Na^+^/K^+^ pump (NKP), NKCC and KCC (red, green, and blue bars, respectively) was calculated as ouabain sensitive; Cl-dependent, bumetanide-sensitive; and Cl-dependent, ouabain and bumetanide-insensitive Rb^+^ influx, respectively. KCC3 AA but not KCC3 WT (results not shown) induction causes a potent increase in flux through KCC3, which is accompanied by inhibition of NKCC and stimulation of NKP. The effects of KCC3 AA induction on NKCC and NKP are abolished when extracellular Na^+^ is replaced with NMDG. *n* = 6 individual determinations from a single experiment, total number of experiments *N* = 2. ^#^*p* < 0.05; ^*^p < 0.005, *n* = 12, data represent the mean ± SEM values. Two-sample *t*-test was employed to determine the statistical significance of the differences between Na^+^ and MDG, as indicated.

Analysis of the ouabain-sensitive Rb^+^ uptake component revealed induction of KCC3 AA triggered a ~3–4 fold increase in NKP activation in 5 min (Figure 2B) when compared with NKP activity upon induction of KCC3 WT (Figure 2A, KCC3 WT 23.2 ± 1.0 and KCC3 AA 87.7 ± 12.9 nmol/mg protein − 5 min), consistent with the known 3–4 fold stimulation of NKP flux to its V_max_ by intracellular Na^+^ and extracellular K^+^ site saturation (reviewed in Lauf and Adragna, 2012). This became further evident when extracellular Na^+^ replacement by NMDG eliminated NKP activity (Figure 3). No changes were observed in K_i_ content between non-induced and induced KCC3 WT cells under the same conditions for KCC3 AA cells (data not shown). These data show that prevention of KCC3 Thr991/Thr1048 inhibitory phosphorylation results in constitutive KCC3a activity that is accompanied by concurrent NKCC1 reversal (i.e., outward Rb^+^ efflux through NKCC1) and NKP stimulation.

### KCC3 Thr991/Thr1408 dephosphorylation triggers significant Cl-dependent and Cl-independent K_i_ loss

**Figure 4:**
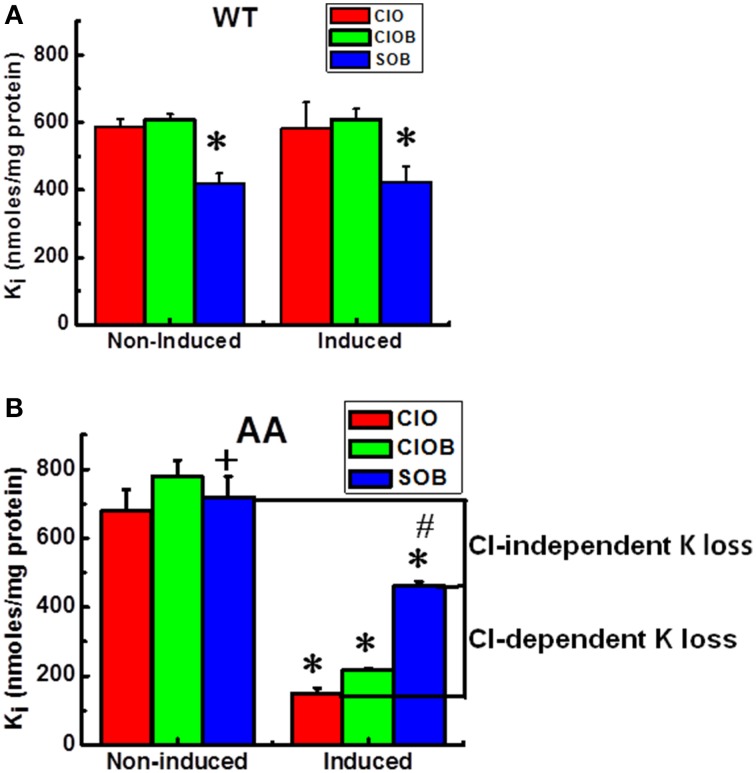
**Constitutive KCC3 Thr991/Thr1048 dephosphorylation elicits rapid and potent Cl-dependent and Cl-independent K_i_ loss**. K_i_ after flux in 10 mM RbCl with ouabain (ClO); ouabain + bumetanide (ClOB); and in 10 mM RbS with ouabain + bumetanide (SOB) in the indicated cell lines [**A** (WT), **B** (AA)] before and after induction with doxycyline. Induction of KCC3 AA results in a large decrease in K_i_ via K^+^ efflux through KCC3. Use of the Cl-free medium SOB partially reduces the magnitude of this K_i_ decrease, indicating that there are both Cl-dependent and Cl-independent components of the K_i_ decrease stimulated by KCC3 AA induction. Cl-dependent K^+^ loss in induced cells was calculated as the K_i_ content in SOB—that in ClO, whereas the Cl-independent K^+^ loss was calculated as the difference in K_i_ content in SOB between non-induced and induced cells. Flux time, 5 min; *n* = 9 individual determinations from a single experiment for WT and *n* = 4 for AA cells. Total number of independent experiments *N* = 4 for WT and *N* = 2 for AA cells. There was a statistically significant difference between groups as determined by Kruskal–Wallis One-Way ANOVA for Non-induced vs. Induced (ClO, ClOB, and SOB) for WT [*F*_(5, 48)_ = 21.55] and AA [*F*_(5, 18)_ = 36.97], *p* < 0.0005, *n* = 54 and 24, respectively. These differences were confirmed by paired *t*-test for (ClOB vs. SOB) in Non-induced and Induced WT, ^*^p < 0.0005, and AA, +*p* < 0.05 (Non-induced) and ^*^p < 0.0005 (Induced); whereas the comparison between Non-induced vs. Induced AA was ^*^p < 0.0005 for ClO and ClOB, and ^#^*p* < 0.005 for SOB. Data represent the mean ± SEM values.

Because intracellular ions and water are determinants of cell volume, any inward ion flux study would be incomplete without considering the fate of the intracellular counter-ion content, in our case K_i_. Furthermore, as opposed to the Rb^+^ influx data shown above, KCC-mediated transport actually results in K^+^ loss (efflux) and requires Cl^−^. Figure 4 shows K_i_ in KCC3 WT (Figure 4A) and KCC3 AA (Figure 4B) cells measured after exposure to Cl^−^ or S media in conditions of NKP inhibition by ouabain, and NKCC inhibition by ouabain + bumetanide, in both non-induced and induced cells. Note that in KCC3 WT cells, there was a 30 % loss of K_i_ in Cl-free medium (601 ± 5.2 in ClOB vs. 417 ± 10.7 in SOB, i.e., 31.5 ± 1.5 % in non-induced and 609 ± 10.6 in ClOB and 423 ± 15.9, i.e., 30.6 ± 1.8 % in induced WT cells, *n* = 9 individual determinations for all the conditions).

Because this K_i_ loss occurred in S but not in Cl^−^ media, it could not have occurred via KCC3, and instead appeared to involve an unidentified K^+^ conductive route, such as a selective K^+^ channel, non-selective cation channel, or transporter. Surprisingly, induction of KCC3 AA triggered a ~75 % K_i_ loss in Cl^−^ media, 50 % of which was recovered by incubating the cells in S medium (see Figure 4B, compare ClO in non-induced and induced cells, and ClO with SOB in induced cells, labeled as Cl-dependent K^+^ loss), indicating inhibition of a Cl-dependent K_i_ loss mechanism presumably through NKCC and KCC3. This Cl-dependent K_i_ loss through NKCC and KCC3 increased as a function of time, further depleting the cells of K^+^ (data not shown), and was inhibited by replacing extracellular Rb^+^ with K^+^, and 2 mM furosemide, an inhibitor of KCC activity (data not shown). In contrast, the remaining 50 % K_i_ loss was Cl-independent (see Figure 4B, compare SOB in non-induced and induced cells).

The Cl-dependent and Cl-independent K_i_ loss triggered by KCC3 AA in isotonic conditions has not been previously reported. The Cl-dependent K^+^ loss that presumably occurs via KCC3 (see above), was matched by a significant increase in Cl-dependent Rb^+^ uptake (Figure 1).

To explore mechanisms contributing to the Cl-independent K_i_ loss, we performed ion replacement studies and tested inhibitors of several different candidate transport systems (see Supplementary Table 3). KCC3 AA-induced Cl-independent K_i_ loss was unaffected by the following: Changes in extracellular Na^+^ (data not shown); TEA (2 mM) and clofilium (100 μM), inhibitors of multiple K^+^ channels (Iwatsuki and Petersen, 1985; Arena and Kass, 1988; Gough and El-Sherif, 1989); Ruthenium Red (1 μM), a non-selective inhibitor of Ca^2+^ conductance; BAPTA (100 μM), an intracellular Ca^2+^ chelator (Tsien, 1980; Tymianski et al., 1994); EDTA (1 mM), an extracellular Mg^2+^/Ca^2+^ chelator; and RN-1734 (30 μM), a TRPV4 channel blocker (Vincent et al., 2009; Vincent and Aj Duncton, 2011) (data not shown and summarized in Supplementary Table 3).

### Cl-independent K_i_ loss elicited by KCC3 Thr991/Thr1048 dephosphorylation is sensitive to the VRAC inhibitor DCPIB

**Figure 5:**
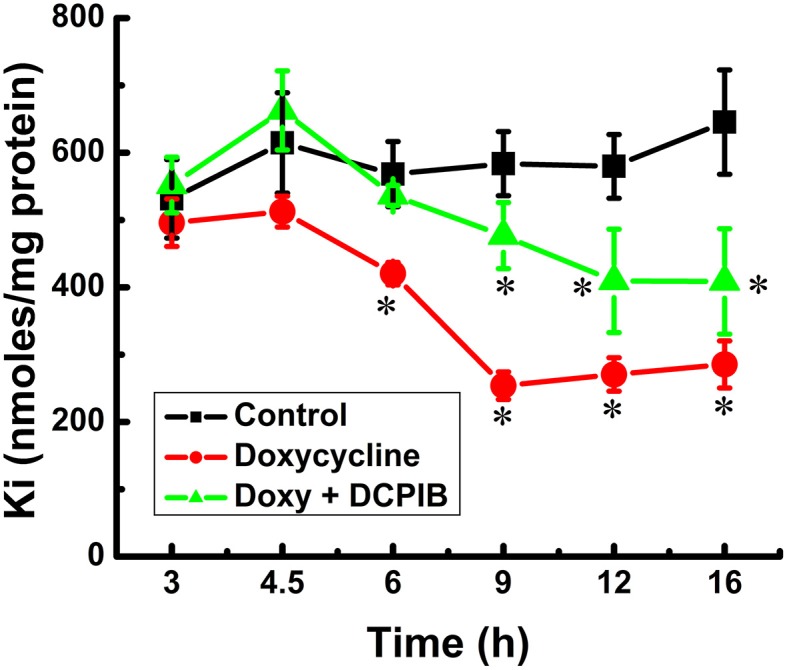
**Constitutive KCC3 dephosphorylation elicits rapid and potent cellular K_i_ loss, which is time-dependent and partially sensitive to DCPIB**. K_i_ content was measured as described in Materials and Methods during induction with doxycycline (1.0 μg/ml) and in the presence and absence of DCPIB (50 μM), an inhibitor of volume regulated Cl^−^ channels (VRACs) and intermediate K^+^ channels. Black squares, Control, i.e., no doxycycline or DCPIB; red circles, doxycycline alone, and green triangles, doxycycline + DCPIB. Time points where there was a significant change were compared vs. the Control. *n* = 8 individual determinations. Total number of independent identical experiments *N* = 2. ^*^p < 0.001; data represent the mean ± SEM values.

**Figure 6:**
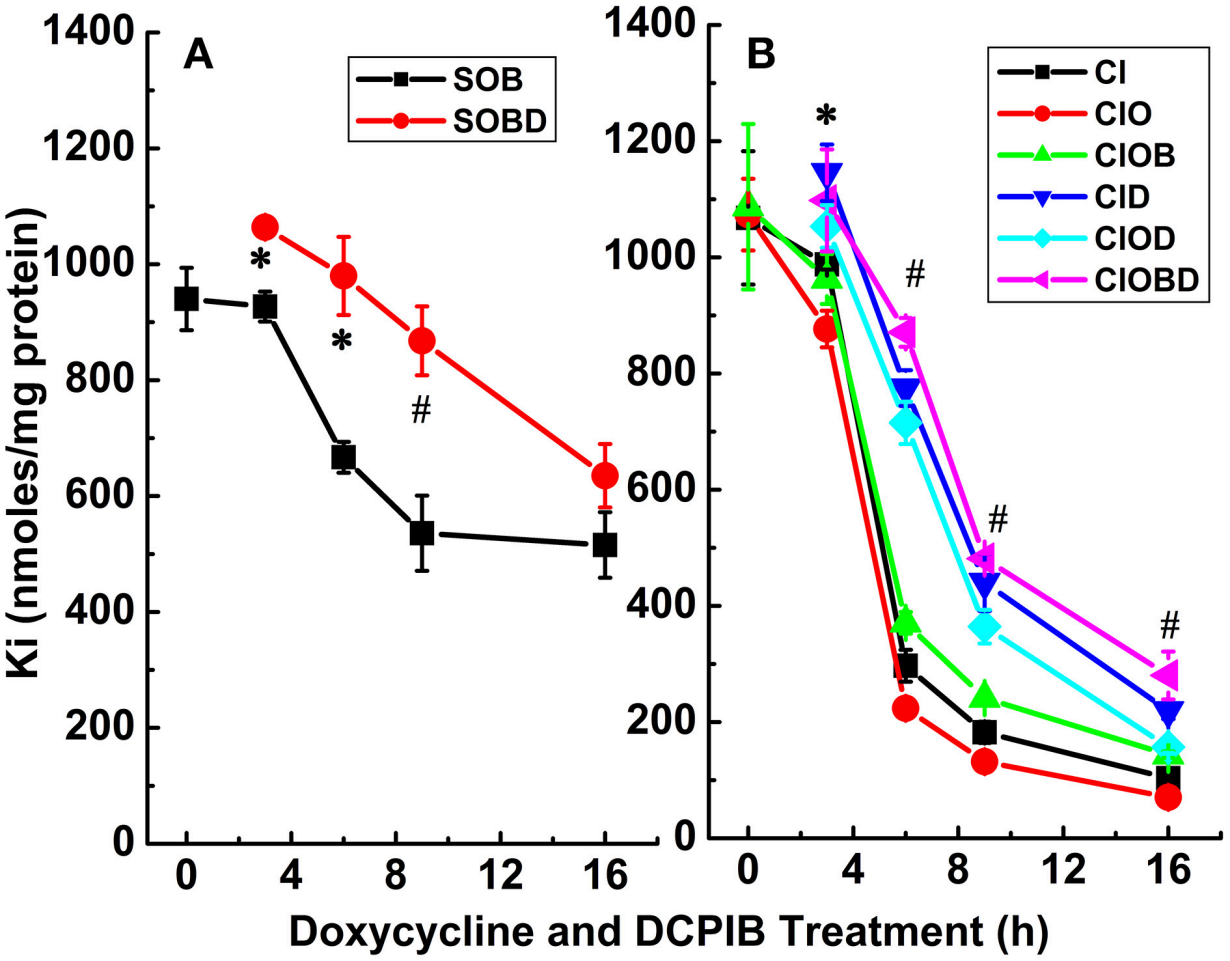
**Constitutive KCC3 Thr991/Thr1048 dephosphorylation elicits rapid and potent cellular K_i_ loss which is time-dependent and partially sensitive to DCPIB**. K_i_ content was measured as described in Materials and Methods during induction with doxycycline (1 μg/ml) and in the presence and absence of DCPIB (50 μM). **(A)** K_i_ content in Cl-free medium, sulfamate (S) replacement without (SOB, black squares) and with DCPIB (SOBD, red circles). **(B)** K_i_ content in Cl-media ± ouabain (0.1 mM) ± bumetanide (10 μM) without (Cl, ClO and ClOB, black squares, red circles, and green triangles, respectively) and with DCPIB (ClD, ClOD and ClOBD, blue triangles point down, cyan diamonds, and magenta triangles point left, respectively). Mean ± SE, *n* = 3–6 individual determinations. **(A)** For SOB vs. SOBD, there was a statistically significant difference between groups as determined by One-Way ANOVA [*F*_(8, 21)_ = 51.65, *p* < 0.0005, *n* = 30], and confirmed by paired *t*-test, ^*^p ≤ 0.01 at 3 and 6 h and ^#^*p* ≤ 0.005 at 9 h. **(B)** For Cl, ClO, ClOB vs. ClD, ClOD, ClOBD, respectively, there was a statistically significant difference between groups as determined by Kruskal–Wallis One–Way ANOVA [*F*_(8, 21)_ = 64.42, 77.64, and 45.44, *p* < 0.0005, *n* = 30], and confirmed by paired *t*-test, ^*^p < 0.05 at 3 h and ^#^*p* < 0.005 at 6 and 9 h. Data represent the mean ± SEM values. Representative results from 4 similar experiments.

**Figure 7:**
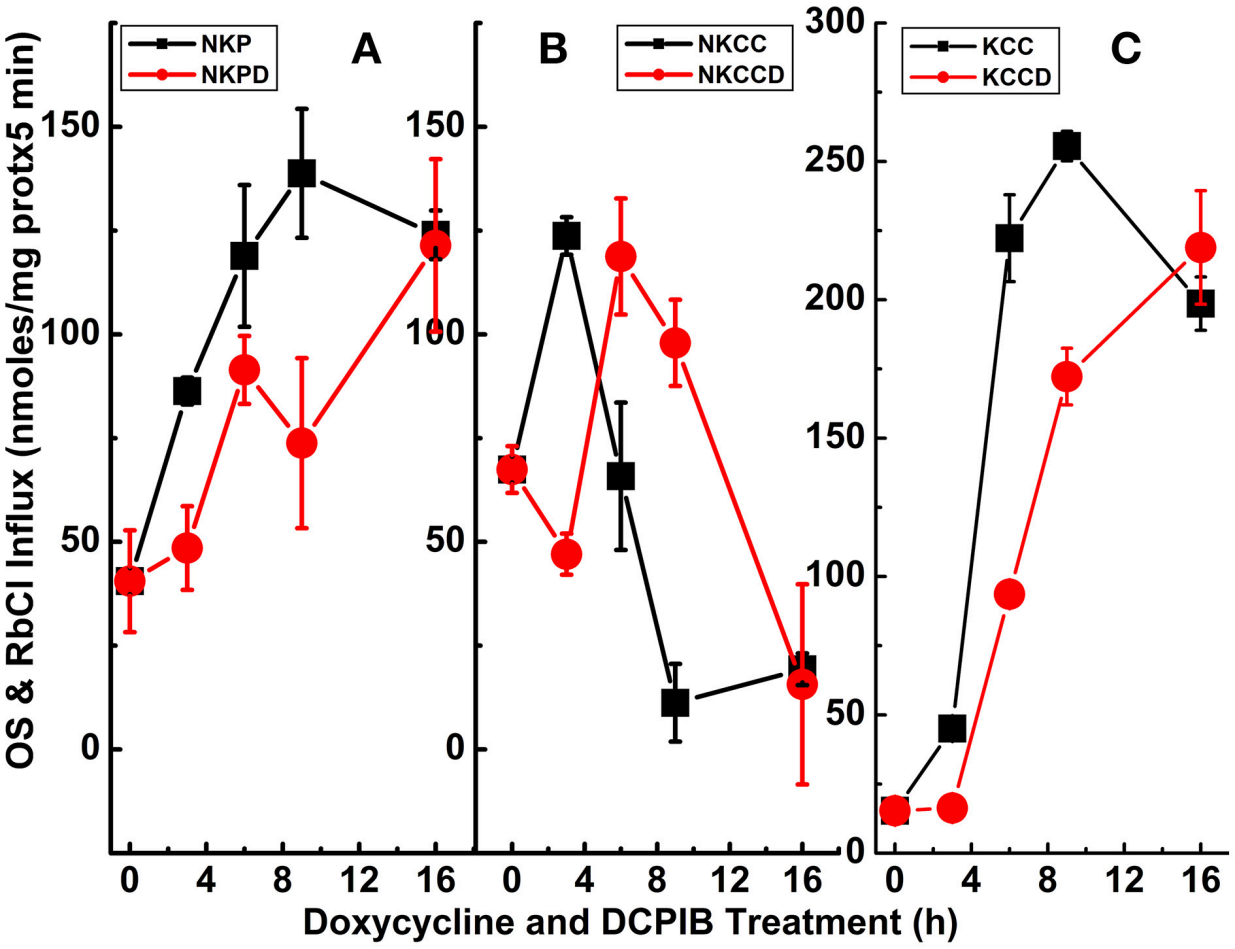
**Constitutive KCC3 Thr991/Thr1048 dephosphorylation elicits potent Rb^+^ influx through ouabain-sensitive (NKP) and Cl-dependent (NKCC and KCC) pathways that are time-dependent and partially sensitive to DCPIB**. Rb^+^ influx was measured simultaneously with the K_i_ contents of Figure 6 and as described in Materials and Methods, during induction with doxycycline (1 μg/ml) and ± DCPIB (50 μM). **(A–C)** Represent NKP, NKCC, and KCC without and with DCPIB (NKPD, NKCCD, and KCCD), respectively. Black squares in the absence and red circles in the presence of DCPIB. *n* = 3–6 individual determinations. There was a statistically significant difference between groups as determined by One-Way ANOVA for NKP vs. NKPD [*F*_(8, 19)_ = 26.06], NKCC vs. NKCCD [*F*_(8, 21)_ = 41.81] and KCC vs. KCCD [*F*_(8, 21)_ = 368.3], *p* < 0.0005, *n* = 30. Data represent the mean ± SEM values. Representative results from 4 similar experiments.

**Figure 8:**
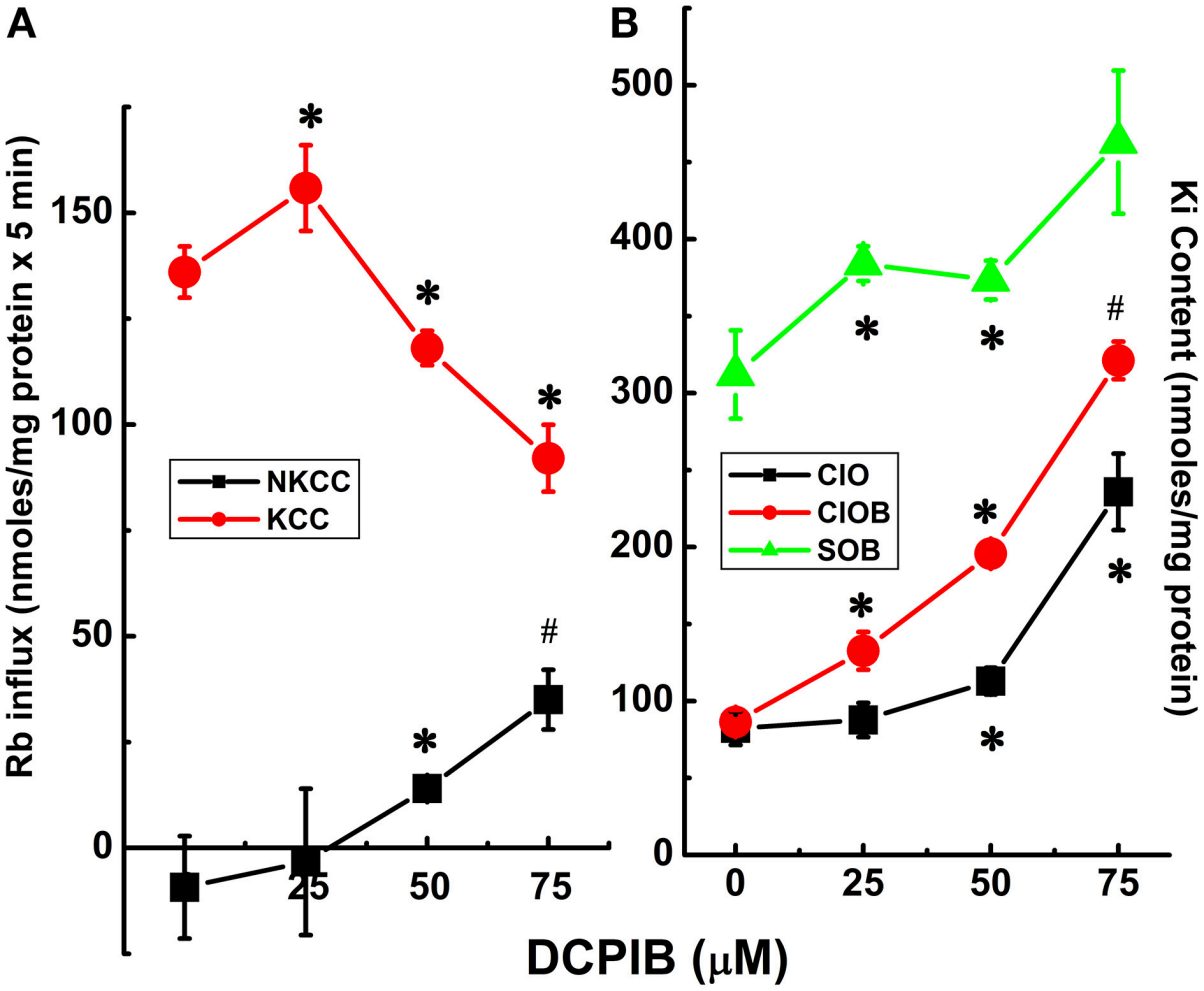
**Cl-dependent Rb^+^ influx and K_i_ content in constitutively dephosphorylated KCC3 cells are DCPIB-sensitive**. Lines indicate doxycycline-induced Rb^+^ influx **(A)** and K_i_ content **(B)**, which were assessed in the following media: Cl^−^ + 0.1 mM ouabain (ClO), Cl^−^ + (ouabain + 10 μM bumetanide) (ClOB), S + (ouabain + bumetanide) (SOB) after induction of KCC3 T991A/T1048A (AA) (see Materials and Methods for details). **(A)** Calculated Rb^+^ flux through NKCC, black squares and KCC, red circles as the Cl-dependent, ouabain-insensitive, bumetanide-sensitive Rb^+^ influx, and Cl-dependent, ouabain and bumetanide-insensitive Rb^+^ influx, respectively. Calculation of NKCC in AA cells after induction may result in a negative or positive value depending on the relative magnitude of the Rb^+^ influx in ClOB with respect to ClO, i.e., CLO − CLOB = + or − NKCC **(A)**; for K_i_ content **(B)** ClO, black squares, CLOB, red circles and SOB, green triangles (see Results and Discussion for further details). Flux time, 5 min; *n* = 4 individual determinations. ^*^p < 0.05 and ^#^*p* < 0.005 as determined by paired *t*-test with respect to 0 μM DCPIB; data represent the mean ± SEM values. Representative results from 4 similar experiments.

Unexpectedly, DCPIB, a blocker of volume-regulated Cl^−^ channel activity (VRAC, or I_Cl−swell_), and known to inhibit K^+^ loss through intermediate K^+^ (IK; K_Ca3.1_) conductance channels (Lauf et al., 2008) caused partial inhibition of K^+^ loss during induction (Figure 5). Furthermore, DCPIB inhibited KCC3 AA-induced Cl-independent K_i_ loss (Figure 6) while inhibiting KCC and NKP and activating NKCC (Figure 7). DCPIB, dose-dependently, also increased NKCC and K_i_ content but inhibited KCC as a function of time when present during the flux (Figure 8).

**Figure 9:**
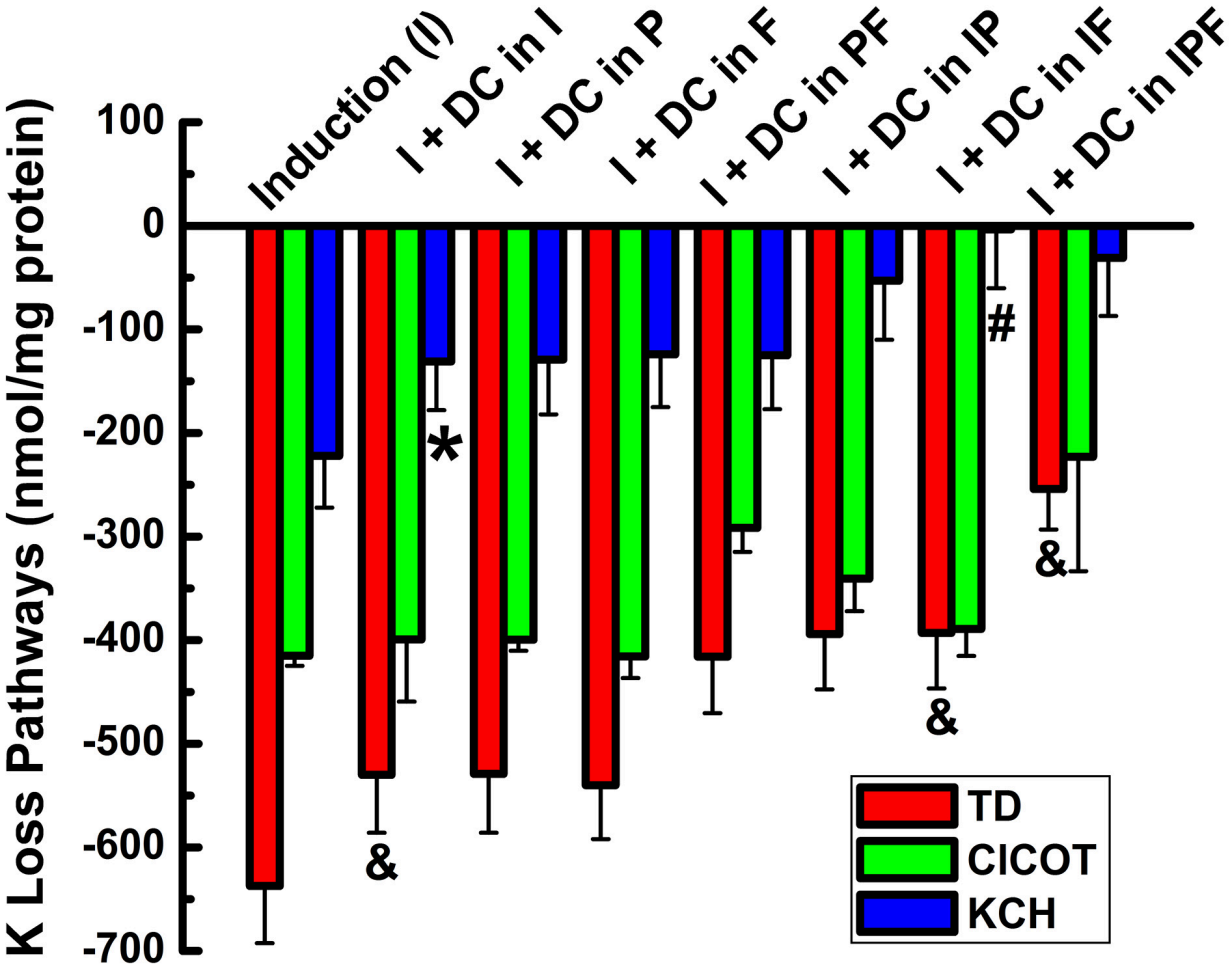
**Cl-independent K_i_ loss trigged by constitutive KCC3 Thr991/Thr1048 dephosphorylation is DCPIB-sensitive**. Bars indicate total doxycycline-induced K_i_ loss (T, red), K_i_ loss through Cl-dependent cotransporters (NKCC + KCC, ClCOT, green), and K^+^ channel-sensitive K_i_ loss (KCH, blue) when DCPIB, an inhibitor of volume regulated Cl^−^ channels (VRACs) and intermediate K^+^ channels, was applied in the induction (I), preincubation (P), and/or flux (F) stages of the ion flux study. DCPIB inhibits Cl-sensitive K_i_ loss. Representative results from a typical experiment done in quadruplicates. Total number of similar experiments, *N* = 4. For T, there was a statistically significant difference between groups as determined by One-Way ANOVA [*F*_(7, 24)_ = 5.15, *p* < 0.001, *n* = 32] or by Kruskal–Wallis One-Way ANOVA [*F*_(7, 24)_ = 4.46, *p* < 0.005, *n* = 32]. There was a statistically significant difference between groups as determined by Kruskal–Wallis One-Way ANOVA [*F*_(8, 27)_ = 12.01, *p* < 0.0005, *n* = 36] for ClCOT but not for KCH [*F*_(7, 24)_ = 1.41, *p* > 0.2, *n* = 32. Data represent the mean ± SEM values. However, there was a statistically significant difference for KCH, ^*^p < 0.05 and ^#^*p* < 0.005; and for TD (&*p* < 0.0005) by paired *T*-Test for the different conditions shown in the figure and described in the text.

**Figure 10:**
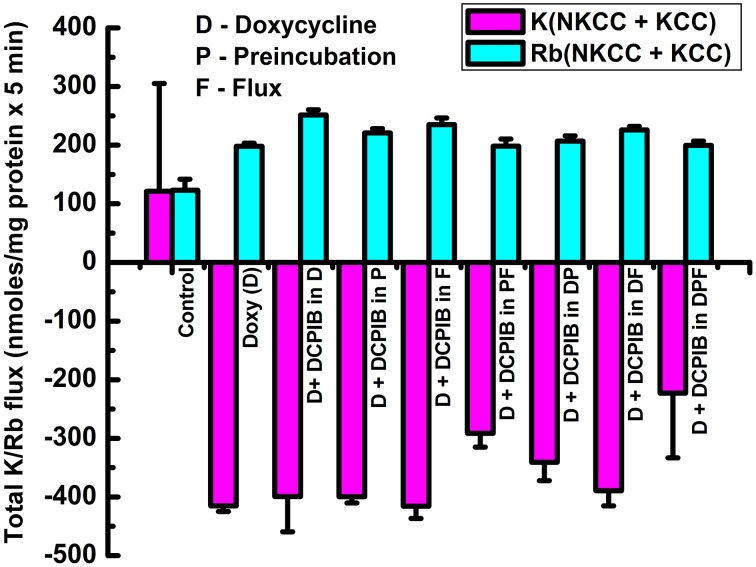
**Cl-dependent Rb^+^ influx and K_i_ loss triggered by constitutive KCC3 Thr991/Thr1048 dephosphorylation is DCPIB-sensitive**. Bars indicate Rb^+^ influx through Cl-dependent cotransporters (NKCC + KCC, cyan) and K_i_ loss (NKCC + KCC, magenta), when DCPIB was applied in the induction (I), preincubation (P), and/or flux (F) stages of the ion flux study. DCPIB inhibits Cl-dependent Rb^+^ influx and K_i_ loss depending on the conditions. Representative results from 4 similar experiments. For Rb^+^ (NKCC + KCC), there was a statistically significant difference between groups as determined by One-Way ANOVA [*F*_(8, 27)_ = 48.75, *p* < 0.0005, *n* = 36]. For K^+^ (NKK + KCC), there was a statistically significant difference between groups as determined by Kruskal–Wallis One-Way ANOVA [*F*_(8, 27)_ = 20.51, *p* < 0.0005, *n* = 36]. Data represent the mean ± SEM values.

To further assess the effect of the VRAC inhibitor on the main transporters studied, DCPIB (50 μM) was applied during the 3 main steps of the Rb^+^ influx protocol; i.e., during KCC3 AA induction (I), pre-incubation (P), flux (F), and in different combinations (IP, IF, and IPF) (Figure 9, Supplementary Tables 1, 2). Three main components for K^+^ loss were defined and were calculated as total KCC3 AA-induced K^+^ loss (T), Cl^−^ cotransporter-dependent K^+^ loss (ClCOT, i.e., through NKCC + KCC, see Figure 10), and K^+^ channel-dependent K^+^ loss (KCH). Figure 9 depicts the total KCC3 AA-induced K^+^ loss (T) through its two components (ClCOT and KCH) in the various conditions.

Strikingly, statistically significant inhibition of KCH by DCPIB was observed when the inhibitor was present during induction (I) alone (I + DC in I) (*p* < 0.05), and complete inhibition when it was present during I + flux (I + DC in IF) (*p* < 0.005), *n* = 4 with respect to I alone, although there was not statistically significant difference between groups (See Figure 9 legend for more details). In the latter condition, K_i_ was maintained at the control level. Similar results were observed when the inhibitor was present during either induction + preincubation (IP), or during induction + preincubation + flux (IPF). These data show KCC3 AA-induced Cl-independent K_i_ loss is DCPIB-sensitive, suggesting dependence on VRAC activity.

The activities of the Cl-coupled cotransporters (NKCC and KCC) were determined by measuring Rb^+^ influx or K^+^ loss under identical conditions as in Figure 9 and the total Rb^+^ influx or K^+^ loss through NKCC + KCC are represented in Figure 10 as their sum for Rb^+^ influx and for K loss. Note that the sum of the two cotransporters was roughly constant for both Rb^+^ influx and K^+^ loss independently of the increasing exposure to DCPIB, and that K^+^ loss was about twice the Rb^+^ influx across the different conditions. This difference may be explained by the driving forces for K_i_ (~140 mM K^+^_i_/0 mM K^+^_o_) and Rb_o_ (0 mM Rb^+^_i_/10 mM Rb^+^_o_). These findings indicate that constitutive dephosphorylation of KCC3 could lead to cell shrinking when VRAC is inhibited by DCPIB.

### Effect of KCC3 Thr991/Thr1048 dephosphorylation on cell volume regulation in hypotonic osmotic stress

**Figure 11:**
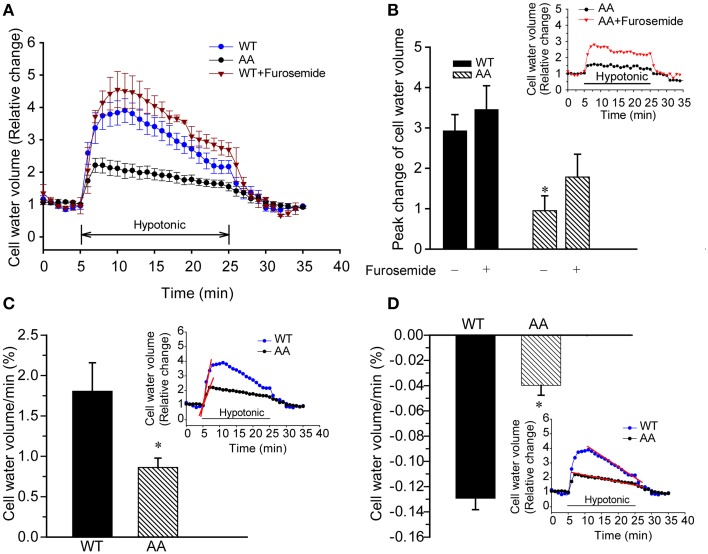
**Constitutive KCC3 Thr991/Thr1048 dephosphorylation reduces acute cell swelling in response to hypotonic osmotic stress. (A)** Representative relative change in cell volume after hypotonic swelling in KCC3 WT and KCC3 AA cells (see Materials and Methods for details). Cells were exposed to isotonic HEPES-MEM (310 mOsm/kg H_2_O), followed by hypotonic HEPES-MEM (150 mOsm/kg H_2_O) for 20 min, and subsequently isotonic HEPES-MEM for 5 min. For drug treatment, cells were pre-incubated with 2 mM furosemide for 15 min prior to the osmotic stress. Furosemide was present throughout the experiment. **(B)** Summary data of cell volume increase. Inset shows representative traces of relative change in cell water in KCC3 AA cells in presence or absence of furosemide. Data are means ± SE. *n* = 5, ^*^P < 0.05 vs. WT control. *P* = 0.1 WT + furosemide vs. AA + furosemide. **(C)** Rate of cell volume change. Bar graphs represent the rate constants from the slopes (red line) calculated by fitting a linear regression to the cell water volume data (relative change) during the initial swelling response (5–6 min, inset: slopes in red). ^*^p < 0.05 vs. WT. **(D)** Regulatory volume decrease (RVD). Bar graphs represent the rate constants from the slopes (red line) calculated by fitting a linear regression to the cell volume relative change data during 10–24 min of cell volume recovery (inset: slopes in red). Data are means ± SE. *n* = 5 experiments. ^*^p < 0.05 vs. WT.

Given the potent Cl-dependent and Cl-independent K_i_ loss induced by KCC3 AA, we speculated these cells might exhibit different swelling properties compared to those expressing KCC3 WT. We therefore examined cell volume changes and RVD in response to hypotonic challenge in KCC3 WT and KCC3 AA cells (see Materials and Methods for details). At baseline, in isotonic conditions, both cell lines exhibited similar cell volumes (Figure 11A). However, hypotonic conditions [HEPES-MEM (150 mOsm/kg H_2_O)] elicited a ~3.2 ± 0.5-fold increase in cell volume (peak values) in KCC3 WT cells, compared to significantly less swelling in KCC3 AA cells [1.2 ± 0.3-fold increase in cell volume (peak values)] (Figures 11A,B). Two phases of cell volume changes were further analyzed in Figures 11C,D. First, KCC3 WT cells showed a sharp swelling increase upon hypotonic challenge (at a rate of 1.8 ± 0.3 % cell volume/min); in contrast, KCC3 AA cells exhibited a significantly slower rate (1.05 ± 0.2 % cell volume/min). Secondly, KCC3 WT cells triggered RVD at a rate of 0.13 ± 0.01 % cell volume/min (Figure 11D) and recovered to ~50 ± 0.2 % of their original volume in ~20 min; in contrast, KCC3 AA cells exhibited a slower RVD rate (0.04 ± 0.007 % cell volume/min) (Figure 11D). These data suggest that constitutive KCC3 Thr991/Thr1048 dephosphorylation renders cells less prone to acute swelling in hypotonic osmotic stress.

## Discussion

Hypotonic cell swelling triggers RVD to maintain cell volume, resulting in K^+^ and Cl^−^ (and obligated water) efflux via the activation of K^+^ channels, VRACs, and KCC3 (Hoffmann and Dunham, 1995). KCC3 Thr991/Thr1048 dephosphorylation accompanies cell swelling (Rinehart et al., 2009; De Los Heros et al., 2014), but the cellular physiology of this event has not been systematically examined. We show constitutive dephosphorylation of KCC3a Thr991/Thr1048, modeled via alanine substitution at the involved residues (preventing phosphorylation), is a potent mechanism of stimulating KCC3a activity, resulting in a rapid (<10 min) and significant (>90%) reduction in K_i_ via both Cl-dependent and Cl-independent (and DCPIB-sensitive) pathways. These effects, along with the associated inhibition of NKCC1 activity and increase in NKP activity, render cells less prone to acute swelling in hypotonic osmotic stress. Our studies with ^85^Rb^+^ and ^39^K^+^ under zero-trans conditions used here and developed several decades ago (Adragna et al., 1994; Adragna and Lauf, 1998), are in agreement with previous studies utilizing radioactive ^86^Rb^+^ under steady state (Rinehart et al., 2009; De Los Heros et al., 2014), and corroborate and extend earlier work demonstrating the potency of KCC3 Thr991/Thr1048 dephosphorylation for transporter activation. Furthermore, and in contrast to previous studies, our findings demonstrating an important role of the KCC3 Thr991/Thr1048 switch as a key regulator of K_i_ content and cell volume in response to hypotonic stress are entirely novel.

In isotonic conditions, KCC3a is robustly phosphorylated at Thr991/Thr1048 and functionally inactive. In these conditions, and consistent with previous findings measuring unidirectional transport with radioactive ^86^Rb^+^ under steady state (Rinehart et al., 2009; De Los Heros et al., 2014), we found genetic prevention of KCC3 phosphorylation at these sites elicited up to a >25-fold increase in transporter activity relative to wild type KCC3. This constitutive KCC3 activity was associated with an inhibition (and subsequent reversal) of NKCC1 activity. The negative values of NKCC1 activity in tested conditions (Figure 1A) likely represent Rb^+^ leak via NKCC1 in the absence of bumetanide, since bumetanide in these conditions resulted in an increase in intracellular Rb^+^ (ClO < ClOB). Constitutive KCC3 activity was also associated with increase in the ouabain-sensitive Rb^+^ uptake component, consistent with increased NKP activity. Extracellular Na^+^ replacement with NMDG eliminated NKP activity (Figure 3), suggesting constitutive KCC3 activity increases cellular Na^+^ entry (which maximally activates NKP in Na^+^ but not in Na-free medium). These data reveal a close functional association between KCC3a and NKCC1/NKP in the context of KCC3a activation, and suggest that attempts to “specifically” inhibit or activate individual CCCs (e.g., blocking NKCC1 or activating KCC2/3) will have effects on other functionally related transport systems that aim to maintain a steady state in ion content and cell volume.

KCC3 AA induction was also accompanied by a rapid and significant K_i_ loss. About 50 % of this K_i_ loss was recovered in Cl-free medium. Partial inhibition of K_i_ loss in sulfamate-containing medium indicated that there are two components to this K_i_ loss: a Cl-dependent and a Cl-independent K_i_ loss. The Cl-dependent K_i_ loss was inhibited by either replacement of extracellular Rb^+^ with K^+^ or furosemide (2 mM), suggesting a dependence on KCC3 and NKCC1-mediated K^+^ efflux. While the Cl-dependent component could thus be explained, the Cl-independent K_i_ loss associated with KCC3 activation required further investigation. We attempted to define this component by inhibiting it with extracellular ion replacement studies or different inhibitors of candidate transport pathways. TEA, clofilium, Ruthenium Red, BAPTA, EDTA, RN-1734, and several other inhibitors had no effect on the Cl-independent K_i_ loss under the conditions tested (Supplementary Table 3). Because of the large K_i_ loss in such a short period of time, a Ca-activated K^+^ channel, such as a BK channel, was thought to be likely involved, as seen in human red blood cells and in vascular smooth muscle, coronary arteries and lens epithelial cells. Thus, 2 mM TEA, which selectively inhibits BK channels, was used, in contrast to 10 mM, which is the concentration used for most Ca-sensitive K^+^ channels of intermediate or smaller conductance. To test the basic idea that Ca^+^ was involved, different approaches, i.e., with inhibitors and chelators, were used, with negative results. Next, clofilium was used as a Ca-independent K^+^ channel blocker, but also with no effect. However, it is possible that BAPTA AM/EDTA under the conditions used in this study may not have effectively depleted other internal sources of Ca_i_.

However, DCPIB, an inhibitor of VRACs (and also intermediate K^+^ conductance channels), completely suppressed the Cl-independent K_i_ loss when used during induction + flux conditions. These results show that constitutive KCC3 activation elicits a rapid and pronounced decrease in K_i_ via Cl-dependent/CCC-mediated pathways, as well as Cl-independent pathways possibly dependent on VRAC activity, such as I_Clswell_ (Voss et al., 2014), or possibly via a yet-unidentified K^+^ conductance that is not inhibited by either TEA or clofilium. In any event, the results obtained with DCPIB seem to shed light on the mechanism by which K^+^ is lost independently of Cl^−^ when the KCC3 AA mutant is overexpressed. Identifying this K^+^ transport pathway will be an interesting topic of detailed future investigation.

The mechanisms by which over-expression of KCC3 AA, resulting in constitutively active K^+^-Cl^−^ cotransport, leads to the associated changes in NKCC and NKP, and K_i_ and cell volume, are summarized in the model in Supplementary Figures 3, 4. Supplementary Figure 3 shows the potential mechanisms accounting for our experimental findings during KCC3 AA *induction* with doxycycline and *preincubation*. These experiments were performed in the presence of 5 mM extracellular K^+^ (K_o_). During these time intervals, KCC3 AA induction (step 1) would trigger KCC-mediated outwardly-directed K^+^ and Cl^−^ loss, and consequently, cell shrinkage. This would *initially* stimulate NKCC1 (step 3), increasing intracellular Na^+^ and Cl^−^ (step 4). The increase in intracellular Na^+^ would stimulate NKP, bringing more K^+^ into cells. The increase in intracellular K^+^ and Cl^−^ (and associated influx of osmotically-obliged water) would then lead to a “rebound” *transient* cell swelling and membrane depolarization (step 5), activating DCPIB-sensitive VRACs (i.e., *Lrrc8-*dependent I_Cl−swell_) (step 6), and indirectly, inducing K_i_ loss (step 7). *During the flux procedure* (Supplementary Figure 4), which measures Rb^+^ influx through KCC, NKCC and NKP in the presence of 10 mM Rb, and K_i_ content at 0 K_o_, the nominally infinite outward gradient for K^+^ causes an outward efflux of K^+^ and Cl^−^ through KCC (step 1), and enhanced entry of Rb^+^ through KCC, NKCC and NKP (step 2). Rb^+^ will enter up to a certain level after which back-flux ensues (Sachs, 1967), *exiting* through NKCC1 (i.e., in a reversal of activity) and blocking its further entry through inwardly directed NKCC1 (step 3). Treatment with DCPIB during induction alone, or induction and pre-incubation, and afterwards during flux, would likely directly inhibit I_Cl−swell_ and indirectly inhibit Cl-sensitive K^+^ loss, as well as the aforementioned pathways for Rb^+^ and K^+^ transport (KCC and NKP), whereas its effect on NKCC1 would appear to be inhibitory or stimulatory depending on the conditions (Figures 6–8).

We examined cell volume changes and RVD in response to acute hypotonic challenge in KCC3 WT and KCC3 AA cells. In these experiments, KCC3 AA cells exhibited significantly less acute cell swelling than KCC3 WT cells in hypotonic conditions. However, KCC3 WT cells exhibited a mildly brisker RVD rate than KCC3 AA cells. These data suggest that constitutive KCC3 activity, achieved via tonic Thr991/Thr1048 dephosphorylation, might render cells less prone to cell swelling in response to hypotonic osmotic stress—particularly during the *early* phases of physiological perturbation. It remains to be determined whether the reduced acute cell swelling in KCC3 AA cells in these conditions is due solely from increased KCC3-mediated ionic efflux, or from the combined effects of this and other volume-regulated transport proteins.

Multiple diseases exhibit dysregulation of cell volume due to aberrant transmembrane ionic transport (Epstein et al., 1995). For example, sickle cell vaso-occlusive events caused by sickle cell dehydration have been associated with hyperactive KCC-mediated cellular K^+^ and Cl^−^ efflux, suggesting KCC inhibition might be a pharmacotherapeutic strategy to prevent sickle cell dehydration and HbS polymerization (Brugnara, 2001). This could be achieved by *promoting* the inhibitory phosphorylation of KCC3 at Thr991/Thr1048 via PP1/PP2a phosphatase inhibition. Conversely, ischemic and traumatic cerebral edema is associated with glial cell swelling due in part to NKCC1-dependent Na^+^, K^+^, and Cl^−^ intracellular accumulation (Su et al., 2002; Lenart et al., 2004; Gagnon et al., 2007). In this context, stimulating KCC3 by *antagonizing* Thr991/Thr1048 phosphorylation would be expected to not only facilitate K^+^ and Cl^−^ efflux and cell shrinkage, but also secondarily decrease the pathological elevation in NKCC1 activity (see Figures 2, 4). The necessity for KCC3-mediated cell volume regulation in the human nervous system has been demonstrated by the autosomal recessive human disease Andermann syndrome, caused by loss-of-function mutations in KCC3, which results in axonal swelling, periaxonal fluid accumulation, and neurodegeneration due to defective RVD (Boettger et al., 2003; Byun and Delpire, 2007).

In elucidating the normal physiological functions of the key phospho-regulatory sites of KCC3, our study suggests that antagonizing the inhibitory phosphorylation of KCC3 at Thr991/Thr1048 would activate KCC3, decrease cellular K^+^ content, and resist acute cell swelling. This could be achieved by inhibiting the WNK-SPAK protein kinase complex (Alessi et al., 2014). SPAK phosphorylates Thr1048 directly, but not Thr991 (Rinehart et al., 2009; De Los Heros et al., 2014), and WNK1 is necessary for both Thr991 and Thr1048 phosphorylation (De Los Heros et al., 2014). Inhibiting the WNK-SPAK pathway would have the added benefit of concurrently decreasing the stimulatory phosphorylation of NKCC1 at Thr212/Thr217, sites required for NKCC1 activity (Thastrup et al., 2012) and pathologically up-regulated in ischemic edema (O'Donnell, 2014). This powerful *coincident* reduction in NKCC1-mediated ion influx and stimulation of KCC3-mediated ion efflux would seem ideally suited to counter acute pathological cell swelling, particularly in glia, in hypotonic swelling conditions as seen in acute hyponatremia from water intoxication. Activation of this KCC3 switch might also be of use in isosmotic swelling, which results from activity-dependent transporter and channel-mediated increases in cellular ionic load (Kahle et al., 2009; Kaila et al., 2014), as in ischemic stroke. Critical to these considerations will be a determination of how constitutive KCC3 Thr991/Thr1048 dephosphorylation might affect long-term cell viability, and CNS and PNS structure and function, in the CNS *in vivo*. We are currently evaluating this in knock-in KCC3 mice carrying double-alanine mutation at Thr991/Thr1048. Lastly, dephosphorylation of the homologous residues in KCC2 (Thr906/Thr1107) might also be a novel method of stimulating KCC2 in diseased neurons to promote Cl^−^ extrusion and restore GABAergic inhibition (Kahle et al., 2013).

### Conflict of interest statement

The authors declare that the research was conducted in the absence of any commercial or financial relationships that could be construed as a potential conflict of interest.

## References

[B1] Adragna N. Di Fulvio M. Lauf P. (2004). Regulation of K-Cl cotransport: from function to genes. J. Membr. Biol. 201, 109–137. 10.1007/s00232-004-0695-6 15711773

[B2] Adragna N. Zhang J. Di Fulvio M. Lincoln T. Lauf P. (2002). KCl cotransport regulation and protein kinase G in cultured vascular smooth muscle cells. J. Membr. Biol. 187, 157–165. 10.1007/s00232-001-0160-8 12029372

[B3] Adragna N. C. (1991). Cation transport in vascular endothelial cells and aging. J. Membr. Biol. 124, 285–291. 10.1007/BF01994361 1787537

[B4] Adragna N. C. Ferrell C. M. Zhang J. Di Fulvio M. Temprana C. F. Sharma A.. (2006). Signal transduction mechanisms of K+-Cl- cotransport regulation and relationship to disease. Acta Physiol. (Oxf.) 187, 125–139. 10.1111/j.1748-1716.2006.01560.x 16734749

[B5] Adragna N. C. Fonseca P. Lauf P. K. (1994). Hydroxyurea affects cell morphology, cation transport, and red blood cell adhesion in cultured vascular endothelial cells. Blood 83, 553–560. 8286751

[B6] Adragna N. C. Lauf P. K. (1998). Role of nitrite, a nitric oxide derivative, in K-Cl cotransport activation of low-potassium sheep red blood cells. J. Membr. Biol. 166, 157–167. 10.1007/s002329900457 9843589

[B7] Adragna N. C. Lauf P. K. (2007). K-Cl cotransport function and its potential contribution to cardiovascular disease. Pathophysiology 14, 135–146. 10.1016/j.pathophys.2007.09.007 17949953

[B8] Alessi D. R. Zhang J. Khanna A. Hochdorfer T. Shang Y. Kahle K. T. (2014). The WNK-SPAK/OSR1 pathway: master regulator of cation-chloride cotransporters. Sci. Signal. 7, re3. 10.1126/scisignal.2005365 25028718

[B9] Altamirano A. A. Breitwieser G. E. Russell J. M. (1988). Vanadate and fluoride effects on Na^+^-K^+^-Cl^−^ cotransport in squid giant axon. Am. J. Physiol. 254, C582–C586. 3354657 10.1152/ajpcell.1988.254.4.C582

[B10] Arena J. P. Kass R. S. (1988). Block of heart potassium channels by clofilium and its tertiary analogs: relationship between drug structure and type of channel blocked. Mol. Pharmacol. 34, 60–66. 2455861

[B11] Boettger T. Hübner C. A. Maier H. Rust M. B. Beck F. X. Jentsch T. J. (2002). Deafness and renal tubular acidosis in mice lacking the K-Cl co-transporter Kcc4. Nature 416, 874–878. 10.1038/416874a 11976689

[B12] Boettger T. Rust M. B. Maier H. Seidenbecher T. Schweizer M. Keating D. J.. (2003). Loss of K−Cl co−transporter KCC3 causes deafness, neurodegeneration and reduced seizure threshold. EMBO J. 22, 5422–5434. 10.1093/emboj/cdg519 14532115 PMC213773

[B13] Brugnara C. (1993). Membrane transport of Na and K and cell dehydration in sickle erythrocytes. Experientia 49, 100–109. 10.1007/BF01989413 8440348

[B14] Brugnara C. (1995). Erythrocyte dehydration in pathophysiology and treatment of sickle cell disease. Curr. Opin. Hematol. 2, 132–138. 10.1097/00062752-199502020-00005 9371983

[B15] Brugnara C. (2001). Therapeutic strategies for prevention of sickle cell dehydration. Blood Cells Mol. Dis. 27, 71–80. 10.1006/bcmd.2000.0366 11358364

[B16] Brugnara C. Bunn H. F. Tosteson D. C. (1986). Regulation of erythrocyte cation and water content in sickle cell anemia. Science 232, 388–390. 10.1126/science.3961486 3961486

[B17] Byun N. Delpire E. (2007). Axonal and periaxonal swelling precede peripheral neurodegeneration in KCC3 knockout mice. Neurobiol. Dis. 28, 39–51. 10.1016/j.nbd.2007.06.014 17659877 PMC2242858

[B18] De Los Heros P. Alessi D. R. Gourlay R. Campbell D. G. Deak M. Macartney T. J.. (2014). The WNK-regulated SPAK/OSR1 kinases directly phosphorylate and inhibit the K+-Cl- co-transporters. Biochem. J. 458, 559–573. 10.1042/BJ20131478 24393035 PMC3940040

[B19] Delpire E. Lauf P. K. (1991). Kinetics of Cl-dependent K fluxes in hyposmotically swollen low K sheep erythrocytes. J. Gen. Physiol. 97, 173–193. 10.1085/jgp.97.2.173 2016578 PMC2216477

[B20] Delpire E. Mount D. B. (2002). Human and murine phenotypes associated with defects in cation-chloride cotransport. Annu. Rev. Physiol. 64, 803–843. 10.1146/annurev.physiol.64.081501.155847 11826289

[B21] Di Fulvio M. Lincoln T. M. Lauf P. K. Adragna N. C. (2001). Protein kinase G regulates potassium chloride cotransporter-3 expression in primary cultures of rat vascular smooth muscle cells. J. Biol. Chem. 276, 21046–21052. 10.1074/jbc.M100901200 11274213

[B22] Dunham P. B. Stewart G. W. Ellory J. C. (1980). Chloride-activated passive potassium transport in human erythrocytes. Proc. Natl. Acad. Sci. U.S.A. 77, 1711–1715. 10.1073/pnas.77.3.1711 6929518 PMC348567

[B23] Epstein F. H. Mcmanus M. L. Churchwell K. B. Strange K. (1995). Regulation of cell volume in health and disease. N. Eng. J. Med. 333, 1260–1267. 10.1056/NEJM199511093331906 7566004

[B24] Gagnon K. B. England R. Delpire E. (2006). Characterization of SPAK and OSR1, regulatory kinases of the Na-K-2Cl cotransporter. Mol. Cell. Biol. 26, 689–698. 10.1128/MCB.26.2.689-698.2006 16382158 PMC1346913

[B25] Gagnon K. B. E. Adragna N. C. Fyffe R. E. W. Lauf P. K. (2007). Characterization of Glial Cell K-Cl Cotransport. Cell. Physiol. Biochem. 20, 121–130. 10.1159/000104160 17595522

[B26] Gough W. B. El-Sherif N. (1989). The differential response of normal and ischaemic Purkinje fibres to clofilium, d-sotalol and bretylium. Cardiovasc. Res. 23, 554–559. 10.1093/cvr/23.6.554 2590928

[B27] Haas M. Forbush B. III. (2000). The Na-K-Cl cotransporter of secretory epithelia. Annu. Rev. Physiol. 62, 515–534. 10.1146/annurev.physiol.62.1.515 10845101

[B28] Hoffmann E. K. Dunham P. B. (1995). Membrane mechanisms and intracellular signalling in cell volume regulation. Int. Rev. Cytol. 161, 173–262. 10.1016/s0074-7696(08)62498-5 7558691

[B29] Hoffmann E. K. Lambert I. H. Pedersen S. F. (2009). Physiology of cell volume regulation in vertebrates. Physiol. Rev. 89, 193–277. 10.1152/physrev.00037.2007 19126758

[B30] Howard H. C. Mount D. B. Rochefort D. Byun N. Dupré N. Lu J.. (2002). The K–Cl cotransporter KCC3 is mutant in a severe peripheral neuropathy associated with agenesis of the corpus callosum. Nat. Genet. 32, 384–392. 10.1038/ng1002 12368912

[B31] Iwatsuki N. Petersen O. (1985). Action of tetraethylammonium on calcium-activated potassium channels in pig pancreatic acinar cells studied by patch-clamp single-channel and whole-cell current recording. J. Membr. Biol. 86, 139–144. 10.1007/BF01870780 2411930

[B32] Jennings M. L. Al-Rohil N. (1990). Kinetics of activation and inactivation of swelling-stimulated K+/Cl-transport. The volume-sensitive parameter is the rate constant for inactivation. J. Gen. Physiol. 95, 1021–1040. 10.1085/jgp.95.6.1021 2373997 PMC2216352

[B33] Jennings M. L. Schulz R. K. (1991). Okadaic acid inhibition of KCl cotransport. Evidence that protein dephosphorylation is necessary for activation of transport by either cell swelling or N-ethylmaleimide. J. Gen. Physiol. 97, 799–817. 10.1085/jgp.97.4.799 1647439 PMC2216490

[B34] Joiner C. H. (1993). Cation transport and volume regulation in sickle red blood cells. Am. J. Physiol. 264, C251–C251. 8447360 10.1152/ajpcell.1993.264.2.C251

[B35] Kahle K. T. Deeb T. Z. Puskarjov M. Silayeva L. Liang B. Kaila K.. (2013). Modulation of neuronal activity by phosphorylation of the K-Cl cotransporter KCC2. Trends Neurosci. 36, 726–737. 10.1016/j.tins.2013.08.006 24139641 PMC4381966

[B36] Kahle K. T. Rinehart J. De Los Heros P. Louvi A. Meade P. Vazquez N.. (2005). WNK3 modulates transport of Cl-in and out of cells: implications for control of cell volume and neuronal excitability. Proc. Natl. Acad. Sci. U.S.A. 102, 16783–16788. 10.1073/pnas.0508307102 16275911 PMC1283843

[B37] Kahle K. T. Simard J. M. Staley K. J. Nahed B. V. Jones P. S. Sun D. (2009). Molecular mechanisms of ischemic cerebral edema: role of electroneutral ion transport. Physiology (Bethesda) 24, 257–265. 10.1152/physiol.00015.2009 19675357

[B38] Kaila K. Price T. J. Payne J. A. Puskarjov M. Voipio J. (2014). Cation-chloride cotransporters in neuronal development, plasticity and disease. Nat. Rev. Neurosci. 15, 637–654. 10.1038/nrn3819 25234263 PMC4294553

[B39] Kregenow F. M. (1971). The response of duck erythrocytes to nonhemolytic hypotonic media evidence for a volume-controlling mechanism. J. Gen. Physiol. 58, 372–395. 10.1085/jgp.58.4.372 5112657 PMC2226034

[B40] Kregenow F. M. (1981). Osmoregulatory salt transporting mechanisms: control of cell volume in anisotonic media. Annu. Rev. Physiol. 43, 493–505. 10.1146/annurev.ph.43.030181.002425 7011197

[B41] Lauf P. (1983). Thiol-dependent passive K/Cl transport in sheep red cells: I. Dependence on chloride and external K+ [Rb+] ions. J. Membr. Biol. 73, 237–246. 10.1007/BF01870538 6864776

[B42] Lauf P. Theg B. (1980). A chloride dependent K+ flux induced by N-ethylmaleimide in genetically low K+ sheep and goat erythrocytes. Biochem. Biophys. Res. Commun. 92, 1422–1428. 10.1016/0006-291X(80)90445-3 7370044

[B43] Lauf P. K. Adragna N. (2012). Properties and transport mechanisms of erythrocytes, in Erythrocytes: Physiology and Pathophysiology, eds Lang F. Foeller M. (London: Imperial College Press), 57–228.

[B44] Lauf P. K. Adragna N. C. (2000). K-Cl cotransport: properties and molecular mechanism. Cell. Physiol. Biochem. 10, 341–354. 10.1159/000016357 11125215

[B45] Lauf P. K. Misri S. Chimote A. A. Adragna N. C. (2008). Apparent intermediate K conductance channel hyposmotic activation in human lens epithelial cells. Am. J. Physiol. Cell Physiol. 294, C820–C832. 10.1152/ajpcell.00375.2007 18184876

[B46] Lenart B. Kintner D. B. Shull G. E. Sun D. (2004). Na-K-Cl cotransporter-mediated intracellular Na^+^ accumulation affects Ca^2+^ signaling in astrocytes in an *in vitro* ischemic model. J. Neurosci. 24, 9585–9597. 10.1523/JNEUROSCI.2569-04.2004 15509746 PMC6730155

[B47] Lew V. L. Bookchin R. M. (2005). Ion transport pathology in the mechanism of sickle cell dehydration. Physiol. Rev. 85, 179–200. 10.1152/physrev.00052.2003 15618480

[B48] Lytle C. Forbush B. III. (1992). The Na-K-Cl cotransport protein of shark rectal gland. II. Regulation by direct phosphorylation. J. Biol. Chem. 267, 25438–25443. 1334094

[B49] O'Donnell M. E. (2014). Blood-brain barrier na transporters in ischemic stroke. Adv. Pharmacol. 71, 113–146. 10.1016/bs.apha.2014.06.011 25307215

[B50] Rinehart J. Maksimova Y. D. Tanis J. E. Stone K. L. Hodson C. A. Zhang J.. (2009). Sites of regulated phosphorylation that control K-Cl cotransporter activity. Cell 138, 525–536. 10.1016/j.cell.2009.05.031 19665974 PMC2811214

[B51] Sachs J. R. (1967). Competitive effects of some cations on active potassium transport in the human red blood cell. J. Clin. Invest. 46, 1433. 10.1172/jci105635 16695928 PMC292889

[B52] Strange K. Denton J. Nehrke K. (2006). Ste20-type kinases: evolutionarily conserved regulators of ion transport and cell volume. Physiology 21, 61–68. 10.1152/physiol.00139.2005 16443823

[B53] Su G. Kintner D. B. Sun D. (2002). Contribution of Na^+^-K^+^-Cl^−^cotransporter to high-[K^+^]_o_- induced swelling and EAA release in astrocytes. Am. J. Physiol. Cell Physiol. 282, C1136–C1146. 10.1152/ajpcell.00478.2001 11940529

[B54] Thastrup J. O. Rafiqi F. H. Vitari A. C. Pozo-Guisado E. Deak M. Mehellou Y.. (2012). SPAK/OSR1 regulate NKCC1 and WNK activity: analysis of WNK isoform interactions and activation by T-loop trans-autophosphorylation. Biochem. J. 441, 325–337. 10.1042/BJ20111879 22032326 PMC3242505

[B55] Tsien R. Y. (1980). New calcium indicators and buffers with high selectivity against magnesium and protons: design, synthesis, and properties of prototype structures. Biochemistry 19, 2396–2404. 10.1021/bi00552a018 6770893

[B56] Tymianski M. Spigelman I. Zhang L. Carlen P. L. Tator C. H. Milton M. P.. (1994). Mechanism of action and persistence of neuroprotection by cell-permeant Ca^2+^ chelators. J. Cereb. Blood Flow Metab. 14, 911–923. 10.1038/jcbfm.1994.122 7929656

[B57] Uyanik G. Elcioglu N. Penzien J. Gross C. Yilmaz Y. Olmez A.. (2006). Novel truncating and missense mutations of the KCC3 gene associated with Andermann syndrome. Neurology 66, 1044–1048. 10.1212/01.wnl.0000204181.31175.8b 16606917

[B58] Vincent F. Acevedo A. Nguyen M. T. Dourado M. Defalco J. Gustafson A.. (2009). Identification and characterization of novel TRPV4 modulators. Biochem. Biophys. Res. Commun. 389, 490–494. 10.1016/j.bbrc.2009.09.007 19737537

[B59] Vincent F. Aj Duncton M. (2011). TRPV4 agonists and antagonists. Curr. Top. Med. Chem. 11, 2216–2226. 10.2174/156802611796904861 21671873

[B60] Voss F. K. Ullrich F. Munch J. Lazarow K. Lutter D. Mah N.. (2014). Identification of LRRC8 heteromers as an essential component of the volume-regulated anion channel VRAC. Science 344, 634–638. 10.1126/science.1252826 24790029

[B61] Zhang J. Lauf P. K. Adragna N. C. (2003). Platelet-derived growth factor regulates K-Cl cotransport in vascular smooth muscle cells. Am. J. Physiol. Cell Physiol. 284, C674–C680. 10.1152/ajpcell.00312.2002 12556360

