# Supplementary material for: Regulated phosphorylation of the K-Cl cotransporter KCC3 is a molecular switch of intracellular potassium content and cell volume homeostasis
Source: Front Cell Neurosci. 2015 Jul 9;9:255. doi: 10.3389/fncel.2015.00255 (PMC4496573; doi:10.3389/fncel.2015.00255)
Supplement: Supplementary file 1 [file DataSheet1.PDF]

Regulated phosphorylation of the K-Cl cotransporter 3 at dual C-terminal threonines is a potent switch of intracellular potassium content and cell volume homeostasis

Norma C. Adragna, Nagendra Babu Ravilla, Peter K. Lauf, Gulnaz Begum, Arjun R. Khanna, Dandan Sun, and Kristopher T. Kahle

SUPPLEMENTARY MATERIALS

SUPPLEMENTARY TABLE 1: Steps, main electrolytes and drugs present during the flux protocol for the indicated figures, and in particular, for those related to identification of the ion transport pathways responsible for KCC3 AA-induced, Cl-independent K_i loss.

Main Electrolytes & Drugs (mM)	KCC3 WT or AA Induction	Preincubation	Flux	Figure #
Na	140	140	140	1-10
K	5	5	0	"
Rb	0	0	10	"
MDG	0	140	140	3
K	5	5	0	1-10
Rb	0	0	10 post-Ind.	7
Ouabain	0	0	0.1	1-10
Bumetanide μM)	0	0	10	1-10
DCPIB (μM)	50	0	0	5-7
"	0	0	0-75	8
"	50 or 0	50 or 0	50 or 0	9-10

SUPPLEMENTARY TABLE 2: Abbreviations and definitions of the media used for the main steps of the flux protocol as described in the text.

Abbreviation	Definition	Abbreviation	Definition
Cl	Cl medium without inhibitors	KCC	K-Cl cotransport/er
ClO	Cl medium + ouabain	NKCC	Na-K-2Cl cotransport
ClOB	Cl medium + ouabain + bumetanide	NKP	Na/K pump
SOB	Sulfamate medium + ouabain + bumetanide	NKPD, NKCCD and KCCD	(NKP, NKCC or KCC) + DCPIB
ClD, ClOD, ClOBD, and SOBD	(Cl, ClO, ClOB, or SOB) + DCPIB	T	Total K loss
NMDG	N-methyl D-glucamine	ClCOT	Cl-dependent cotransporters (NKCC + KCC)
I	Induction	KCH	K channel
P	Preincubatio	RVD	Regulatory volume decrease
F	Flux	RVI	Regulatory volume increase
DC	DCPIB	VRAC	Volume-regulated anion channel
BSS	Balanced salt solution	BSS-NaCl or BSS-NaS	Balanced salt solution in Cl or Sulfamate
BSA	Bovine serum albumin	BSS-(NaCl or -NaS)-BSA	(BSS-NaCl or BSS-NaS) + bovine serum albumin
BSS-RbCl or RbS)-BSA	BSS + (RbCl or RbS) + BSA		

SUPPLEMENTARY TABLE 3: Drugs used in experiments to identify the ion transport pathway responsible for KCC3 AA-induced, Cl-independent K_i loss.

**Drug**	**Description**	**Concentration**	**Extracellular $[Ca^{2+}]$ (mM)**	**Effect on K_i**
TEA	Inhibitor of Ca^{2+}-dependent big conductance (BK) K^+ channels	2 mM	2	None
Clofililum tosylate	Inhibitor of Ca^{2+}-independent K^+ channels	100 µM	2	None
BAPTA-AM	Intracellular Ca^{2+} chelator	100 µM	0	None
EDTA	Extracellular Ca^{2+} chelator	1 mM	0	None
BAPTA-AM/EDTA	Intracellular + extracellular Ca^{2+} chelation	100 µM/1 mM	0	None
RN-1734	TRPV4 channel blocker	30 µM	2	None
Ruthenium Red	Inhibitor of mitochondrial Ca^{2+} uniporter	1 µM	2	None

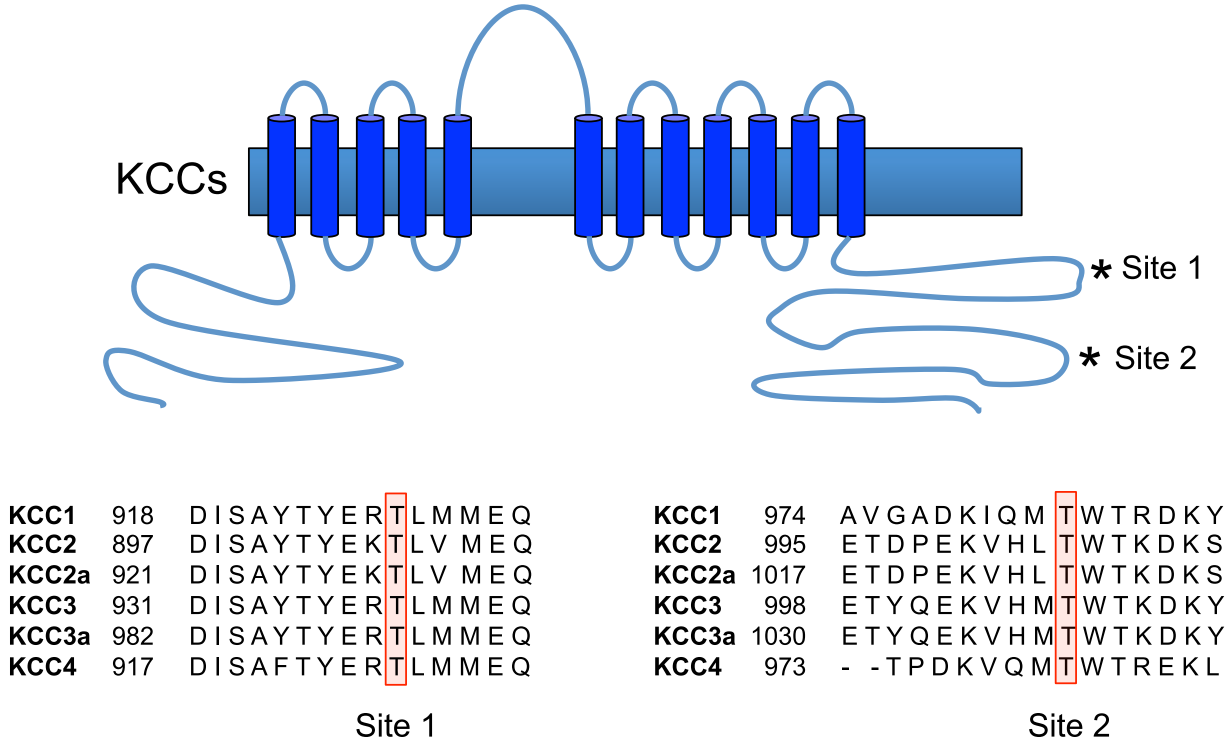

SUPPLEMENTARY FIGURE 1: A conserved inhibitory phosho-motif in the K-Cl contransporters (KCCs) composed of two threonine (Thr) residues in the transporter carboxyl-terminus. Schematic depiction of the two key inhibitory threonine (Thr) phosphorylation sites conserved in all KCC isoforms (KCC1-KCC4). Site 1 in KCC3a is Thr991. Site 2 in KCC3a in Thr1048. In normal isogenic conditions, KCC3a is phosphorylated at both Site 1 and Site 2, and the transporter is functionally inactive (de Los Heros et al., 2014). Hypotonic swelling conditions decreases the inhibitory phosphorylation of KCC3a in association with transporter activation. KCC3 can also be maximally activated, even in normally inhibitory isotonic conditions, by preventing phosphorylation at Thr991/Thr1048, e.g., by genetic alanine substitution at these sites, or promoting PP1/PP2A phosphatase activity (Rinehart et al., 2009a;de Los Heros et al., 2014).

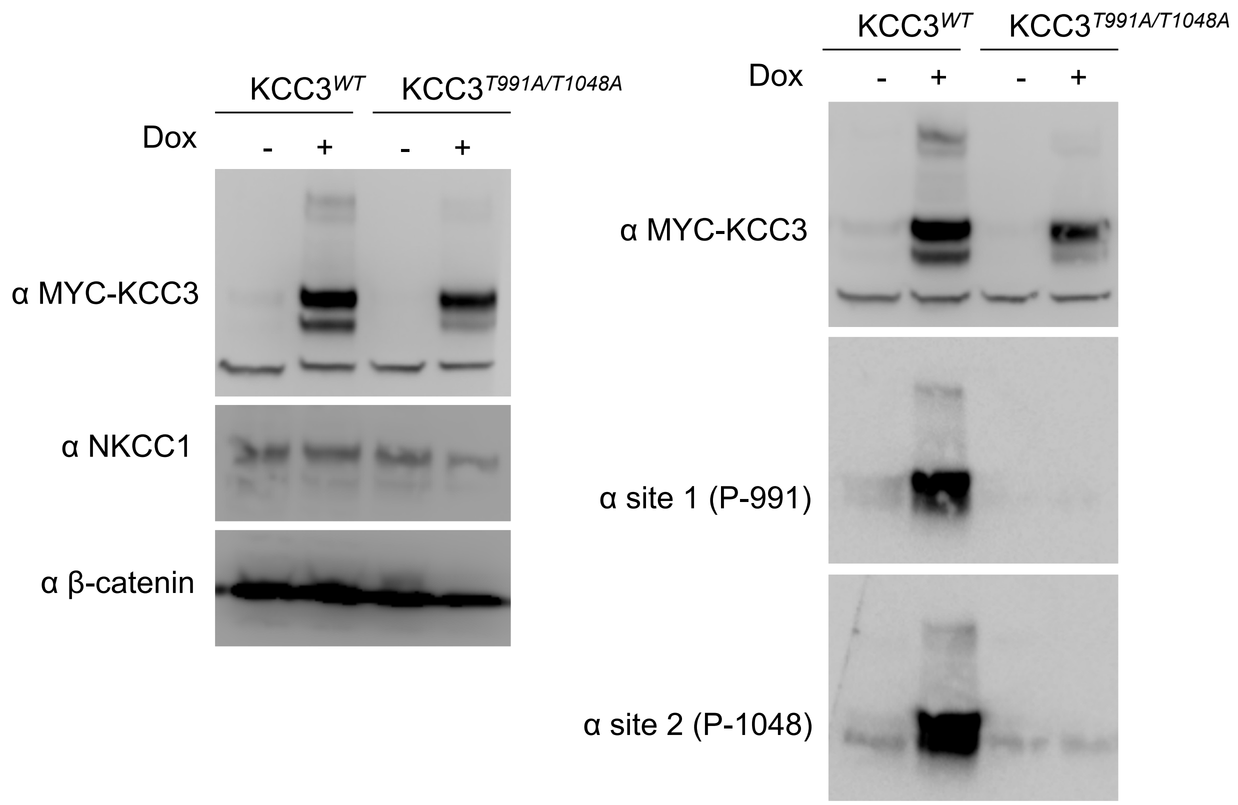

SUPPLEMENTARY FIGURE 2: Creation of an isogenic human epithelial cell line system with inducible expression of wild type (WT) or KCC3 (Thr991Ala/Thr1048Ala). Left panel: Characterization of Flip-in TREX HEK 293 KCC3 wild type (WT) and KCC3 Thr991Ala/Thr1048Ala (AA) isogenic cell lines, harboring doxycycline (dox)-inducible expression of either KCC3 WT or KCC3 AA (see Methods for details of cell line construction). In the absence of dox, cells do not express myc-tagged WT KCC3 or KCC3 AA. However, treatment of cells with dox at concentrations < 1 μM stimulates KCC3 WT or KCC3 AA protein expression in as little as 1.5 h. In isotonic conditions, KCC3 WT but not KCC3 AA is phosphorylated at Thr991 ("site 1") and Thr1048 ("site 2"), as shown using phospho-specific antibodies targeted against these residues (de Los Heros et al., 2014).

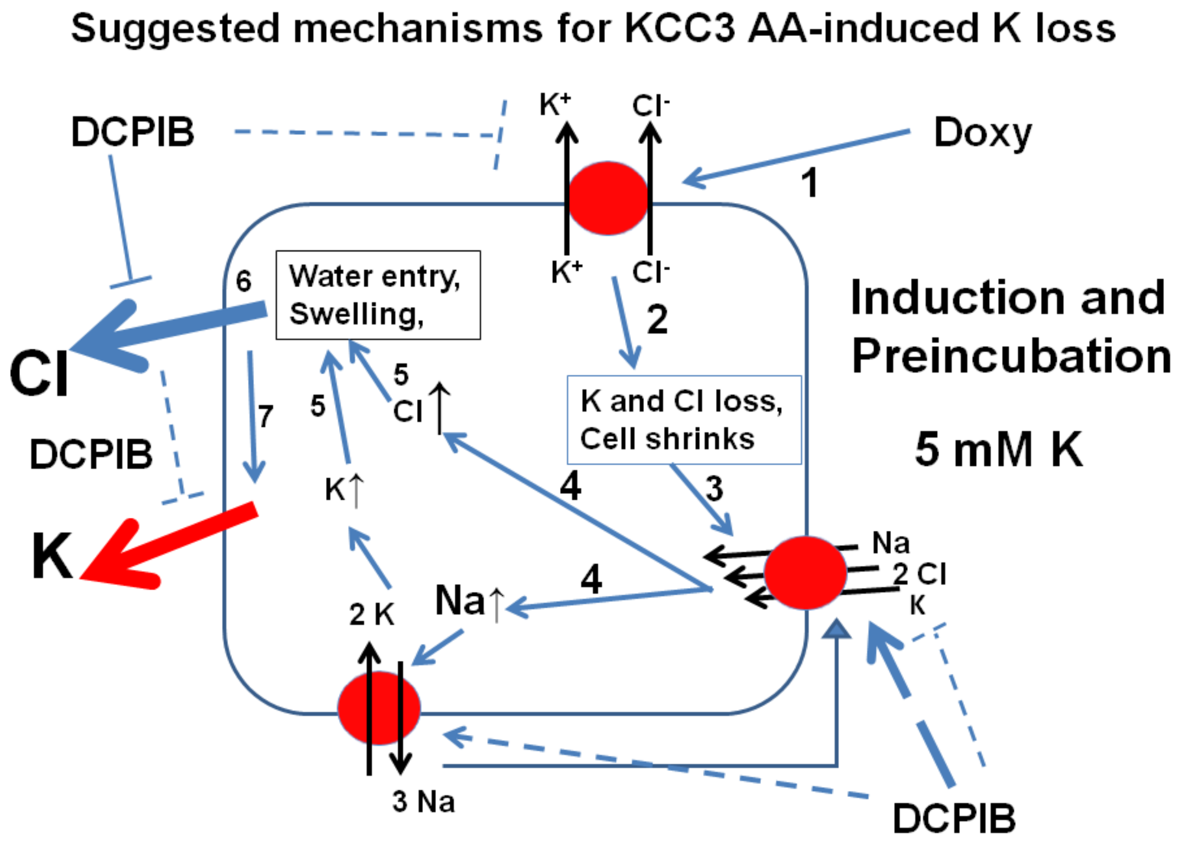

SUPPLEMENTARY FIGURE 3: Suggested mechanism for the effect of constitutive KCC3 activity, achieved via genetic Ala substitution at Thr991/Thr1048, on Rb influx and K^+ loss through NKP, NKCC and KCC during induction and pre-incubation conditions. This model may explain how expression of KCC3 AA leads to an increase of KCC activity, decrease of K_i, increase of NKCC and NKP, and loss of K^+ through swelling and depolarization-induced Cl-coupled K^+ channels during the induction and pre-incubation conditions of our experiments (see Methods for details). Changes in Rb^+ and K_i were determined during induction with doxycycline (1.0 µg/ml) ± DCPIB (50 µM). Both induction and pre-incubation experiments occurred in the presence of 5 mM K^+ in the medium (K_o). During these time intervals, KCC3 AA induction (step one) would trigger KCC-mediated outwardly-directed K^+ and Cl^- loss, and consequently, cell shrinkage. This would *initially* stimulate NKCC1 (step 3), increasing intracellular Na^+ and Cl^- (step 4). The increase in intracellular Na^+ would stimulate NKP, bringing more K^+ into cells. The increase in intracellular K^+ and Cl^- (and associated influx of osmotically-obliged water) would then lead to a "rebound" *transient* cell swelling and membrane depolarization (step 5), activating DCPIB-sensitive VRACs (i.e., *Lrrc8*-dependent $I_{Cl\text{–}swell}$) (step 6), and indirectly, inducing K_i loss (step 7).

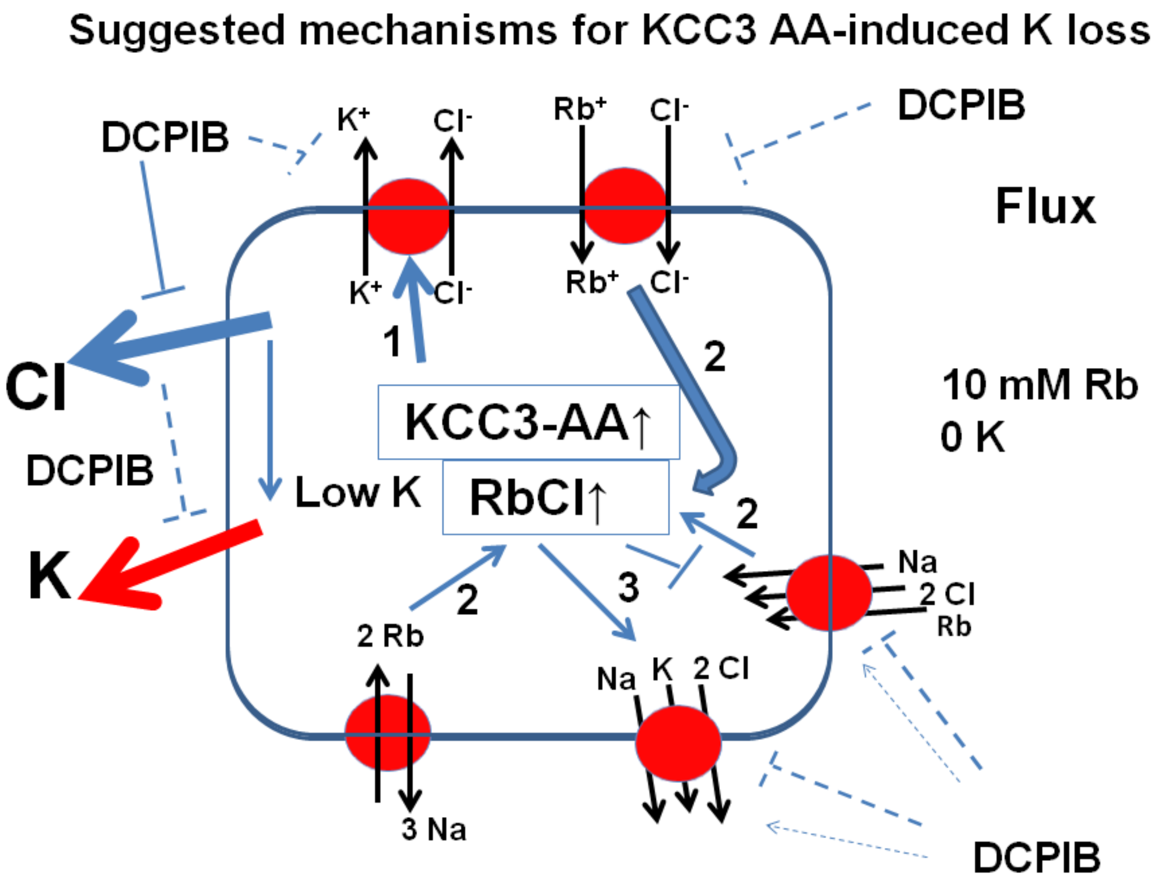

SUPPLEMENTARY FIGURE 4: Suggested mechanism for the effect of constitutive KCC3 activity, achieved via genetic Ala substitution at Thr991/Thr1048, on Rb influx and K^+ loss through NKP, NKCC and KCC in flux conditions. . Prior to flux, cells have been subject to induction with doxycycline (dox) (1.0 µg/ml) ± DCPIB (50 µM) and pre-incubation in the presence of 5 mM K^+ in the medium. For flux measurements, cells are incubated in a buffer containing 10 mM Rb, 0 K, ± DCPIB (0-75 µM), in the absence of dox (since protein expression was already induced). *During the flux procedure*, which measures Rb^+ influx through KCC, NKCC and NKP in the presence of 10 mM Rb, and Ki content at 0 K_o, the nominally infinite outward gradient for K^+ causes an outward efflux of K^+ and Cl^- through KCC (step 1), and enhanced entry of Rb^+ through KCC, NKCC and NKP (step 2). Rb^+ will enter up to a certain level after which back-flux ensues (Sachs, 1967), *exiting* through NKCC1 (i.e., in a reversal of activity) and blocking its further entry through inwardly directed NKCC1 (step 3). Treatment with DCPIB during induction alone, or induction and pre-incubation, and afterwards during flux, would likely directly inhibit $I_{Cl\text{–}swell}$ and indirectly inhibit Cl-sensitive K^+ loss, as well as the aforementioned pathways for Rb^+ and K^+ transport (KCC and NKP), whereas its effect on NKCC1 would appear to be inhibitory or stimulatory depending on the conditions (Figs. 6-8).
